# Nitric oxide-releasing micelles with intelligent targeting for enhanced anti-tumor effect of cisplatin in hypoxia

**DOI:** 10.1186/s12951-021-00989-z

**Published:** 2021-08-16

**Authors:** Yan Chen, Lei Fang, Weixin Zhou, Jinghan Chang, Xiaojuan Zhang, Chuanchuan He, Chen Chen, Ruicong Yan, Yakai Yan, Yao Lu, Chuanrui Xu, Guangya Xiang

**Affiliations:** School of Pharmacy, Tongji Medical College, Huazhong University of Science and Technology, Wuhan, 430030 China

**Keywords:** Hypoxia, Drug resistance, Metastasis, HIF-1α, Cisplatin, Nitric oxide, Chitosan oligosaccharide

## Abstract

**Background:**

Hypoxic tumor microenvironment (TME) promotes tumor metastasis and drug resistance, leading to low efficiency of cancer chemotherapy. The development of targeted agents or multi-target therapies regulating hypoxic microenvironment is an important approach to overcome drug resistance and metastasis.

**Methods:**

In this study, chitosan oligosaccharide (COS)-coated and sialic acid (SA) receptor-targeted nano-micelles were prepared using film dispersion method to co-deliver cisplatin (CDDP) and nitric oxide (NO) (denoted as CTP/CDDP). In addition, we explored the mechanisms by which NO reversed CDDP resistance as well as enhanced anti-metastatic efficacy in hypoxic cancer cells.

**Results:**

Because of the different affinities of COS and SA to phenylboronic acid (PBA) under different pH regimes, CTP/CDDP micelles with intelligent targeting property increased cellular uptake of CDDP and enhanced cytotoxicity to tumors, but reduced systemic toxicity to normal organs or tissues. In addition, CTP/CDDP showed stimulus-responsive release in TME. In terms of anti-tumor mechanism, CTP/CDDP reduced CDDP efflux and inhibited epithelial-mesenchymal transition (EMT) process of tumor by down-regulating hypoxia-inducible factor-1α (HIF-1α), glutathione (GSH), multidrug resistance-associated protein 2 (MRP2) and matrix metalloproteinase 9 (MMP9) expression, thus reversing drug resistance and metastasis of hypoxic tumor cells.

**Conclusions:**

The designed micelles significantly enhanced anti-tumor effects both in vitro and in vivo. These results suggested that CTP/CDDP represented a promising strategy to treat resistance and metastatic tumors.

**Graphic abstract:**

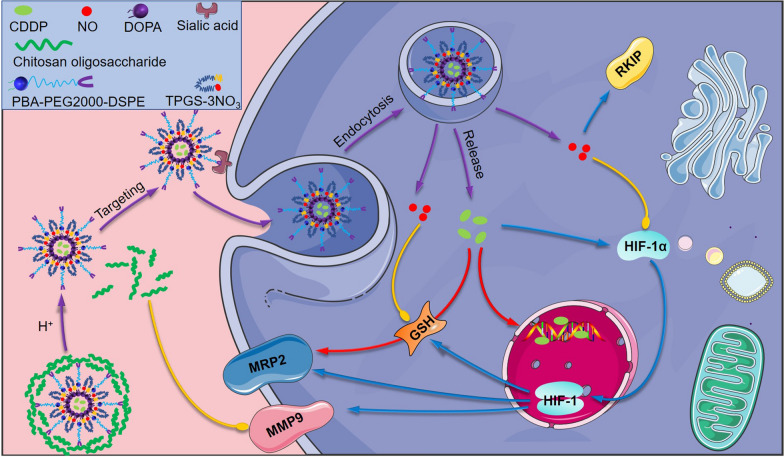

**Supplementary Information:**

The online version contains supplementary material available at 10.1186/s12951-021-00989-z.

## Background

Tumor microenvironment (TME), characterized with hypoxic and acidic conditions, contributes to the multidrug resistance (MDR) and metastasis of tumor [1]. Hypoxia is initiated in the early stage of tumorigenesis [2], and generates a profound impact on the physiological properties of tumors by inducing expression of hypoxia inducible factors (HIFs) in tumor cells [3, 4]. HIF-1 is a heterodimer composed of α and β subunits, and HIF-1α is tightly regulated by hypoxia, oxygen, and reactive oxygen species [5]. Under hypoxic conditions, HIF-1α-dependent ubiquitin–proteasome degradation pathway is blocked, and HIF-1α is able to translocate into the nucleus to form HIF-1 with the β subunit, thereby initiating or enhancing the transcription of effector genes [6].

Numerous studies have shown that HIF-1 plays an important role in hypoxia-induced cancer resistance and metastasis, and the underlying mechanisms mainly include the following pathways: (1) HIF-1 promotes the synthesis of glutathione (GSH) under hypoxia, thereby decreasing the killing effect of reactive oxygen species (ROS) [7, 8]. (2) HIF-1 enhances the expression of ATP-binding cassette (ABC) transporters such as P-glycoprotein (P-gp) and multidrug resistance-associated protein (MRP), which can promote the efflux of chemotherapeutic drugs [9]. (3) HIF-1 can up-regulate anti-apoptotic factors such as Bcl-2, Bcl-xl and NF-κB, and inhibit the expression of pro-apoptotic factors such as BAX, BNIP3 and NOXA, thus promoting tumor cell proliferation [10]. (4) HIF-1 is able to suppress the DNA damage mediated by chemotherapeutic drugs and enhances the MDR of cancer cells [11]. (5) HIF-1 can improve the proliferation of tumor cells by inhibiting mitochondrial activity and occurrence of cell apoptosis and necrosis [12]. (6) HIF-1 is able to increase the expression of matrix metalloproteinases (MMPs), which degrades extracellular matrix (ECM), promotes epithelial-mesenchymal transition (EMT) process, and exacerbates cancer metastasis [13].

In addition to hypoxia, chemotherapy can promote the expression of HIF-1 and further enhance the drug resistance and metastasis of tumor cells [14, 15]. Chemotherapy increases the level of intracellular HIF-1α and induces the high expression of downstream MRP, GSH and MMP9 [16, 17]. For example, cisplatin (CDDP) readily forms complex with GSH after hydrolysis in the cytoplasm, making it unable to enter cell nucleus and bind to the target DNA [18, 19]. The formed complex can be easily pumped out of tumor cell by efflux proteins such as MRP1 and MRP2, resulting in exacerbated drug resistance [20]. Moreover, on the basis of limited inhibitory effect of CDDP to cancer metastasis [21], the hypoxic microenvironment can further promote cancer metastasis by increasing the expression of MMPs.

To sum up, HIF-1α is an important target for drug resistance and metastasis of tumor cells. Nitric oxide (NO) is an endogenous molecule that plays a key regulatory role in physiological processes including neurotransmission, apoptosis, vascular smooth muscle relaxation, immune response and so on [22]. In recent years, the application of NO in cancer treatment has been widely investigated [23]. It has been reported that NO treatment can effectively inhibit the expression of HIF-1α in hypoxic tumor cells [24]. Furthermore, NO is able to directly bind to GSH, thereby reducing the GSH concentration within cancer cells [25]. Therefore, the combination of chemotherapeutic drugs and NO therapy can effectively solve the problem of hypoxic tumor cells prone to resistance and metastasis.

However, NO has extremely short half-life and unstable chemical properties, making it difficult to be delivered directly to the tumor tissues. Therefore, delivery of NO via nitric oxide donors (NOD) is utilized to produce drugs with enhanced stability. In recent years, a variety of NOD have been developed to produce NO in situ, including sodium nitroprusside, nitrosothiols, nitroglycerin, etc. [26–28]. However, most of these NOD are small molecular compounds, which can be easily cleared by the reticuloendothelial system (RES) during blood circulation [29]. In order to prolong the circulation time and improve the pharmacokinetics, researchers have coupled small molecule NOD with dendrimers, liposomes and micelles to prepare NO-releasing nanoparticles [30]. In this study, TPGS-3NO_3_, a polymer with nitrate ester groups was synthesized and used as component to produce nano-micelles capable of releasing NO.

In order to enhance the anti-tumor efficacy, some new functions, such as active targeting and stimuli-responsive drug release were introduced into the design of nano-micelles. Sialic acid (SA), a monosaccharide located at the end of the side chain on cell membrane glycoprotein, is overexpressed on the surface of many malignant and metastatic cancer cells and is an important targeted receptor [31]. Phenylboronic acid (PBA) is able to reversibly combine with 1,2- or 1,3-dihydroxy compounds such as SA and chitosan oligosaccharide (COS) to form phenylboronate ester [32]. Therefore, the modification of PBA ligands on the surface of nano-micelles can endow nanoparticles with active targeting function, which is beneficial to the enrichment and uptake of nano-drugs at the tumor site. The current strategy is to modify PBA directly on the surface of nanoparticles. Kundu et al. prepared PBA-conjugated and pH-responsive ZnO nanoparticles (ZnO-PBA) for the tumor tissue-specific delivery of curcumin [33]. The results showed that ZnO-PBA exhibits a higher cellular uptake in breast cancer cells compared to ZnO NPs, indicating that PBA conjugation facilitates the targeted delivery of curcumin to the SA overexpressed cancer cells. However, normal liver and lung cells also express SA in small amounts on their surface [34], directly modification of PBA ligands will allow nano-micelles to target these tissues and increase side effects while reducing therapeutic effect.

**Fig. 1:**
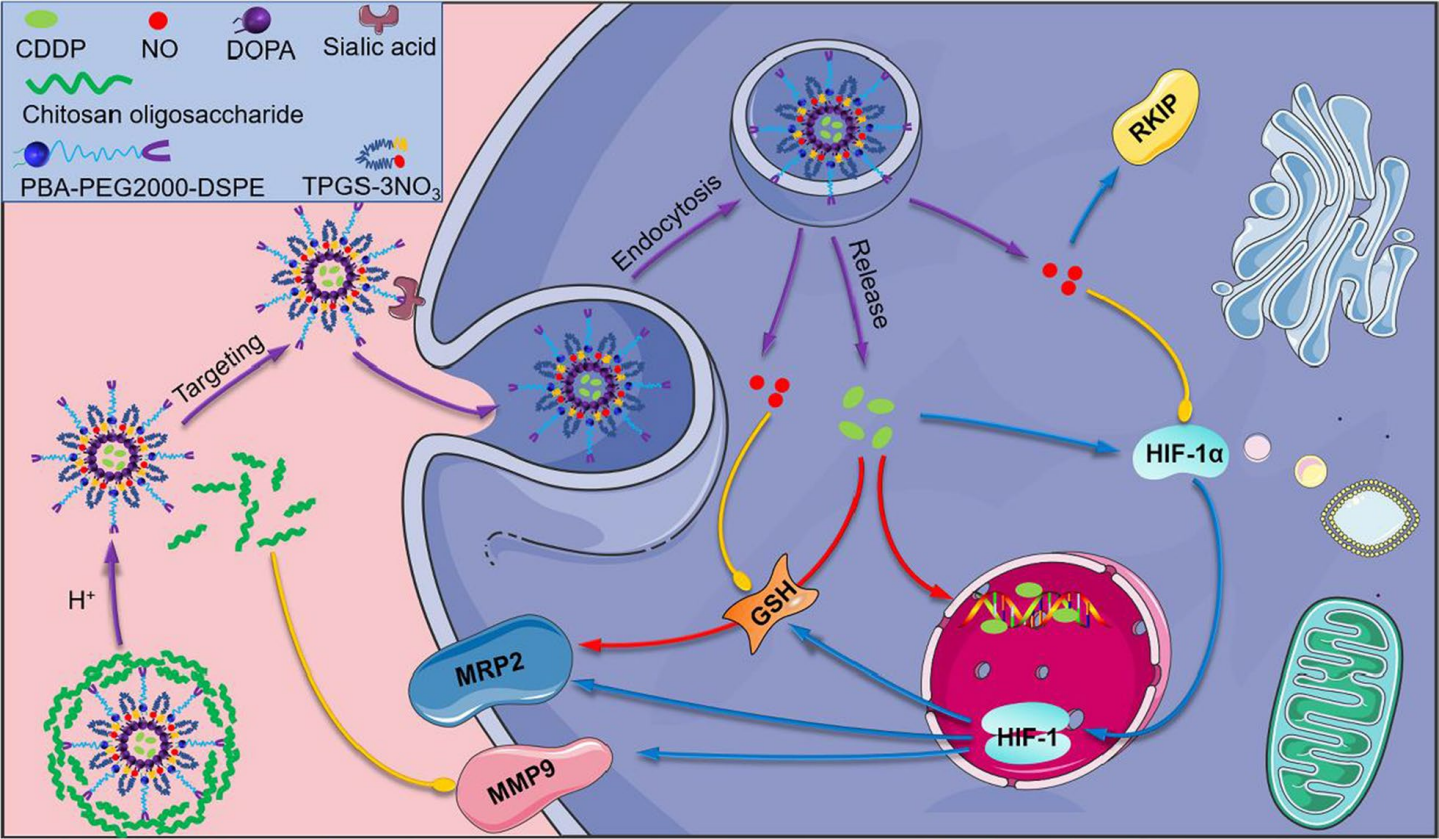
Schematic diagram of CTP/CDDP targeting SA residues, and reversing drug resistance and metastasis of hypoxic cancer cells

In this study, we designed SA receptor-targeted nano-micelles coated with COS for co-delivery of CDDP and NO (denoted as CTP/CDDP) to achieve the TME-responsive uptake and drug release. Due to the different affinities of COS and SA to PBA under different pH conditions, the nano-micelle could intelligently target tumor cells and release the loaded drugs. As illustrated in Fig. 1, PBA ligands on the surface of nano-micelles were enclosed with COS in the neutral environment, so the nano-micelles were avoided being taken up by normal cells. While in the acidic microenvironment of tumor, the outer COS coating would be broken down and consequently PBA ligands were exposed and then recognized by the SA receptors of tumor cells. Notably, the COS could inhibit the metastasis of tumor cells simultaneously [35]. Moreover, the drug release behavior of nano-micelles was TME responsive. In terms of anti-tumor mechanism, CTP/CDDP could directly or indirectly inhibit the expression of HIF-1α, GSH and MRP2, reduce the efflux of CDDP and alleviate the chemotherapeutic drug resistance in tumor cells. In addition, CTP/CDDP enhanced the expression of RKIP and inhibited the HIF-1α/MMP9 pathway by releasing NO and COS in hypoxic cells, thus inhibiting the EMT process of tumor and enhancing the anti-metastatic effect of chemotherapeutic drugs.

## Materials and methods

### Materials

D-α-tocopherol polyethylene glycol 1000 succinate (TPGS), dimethyl sulfoxide-d6 (DMSO-d6), Igepal CO-520, CDDP and coumarin 6 (C6) were supplied by Sigma-Aldrich Chemical Co. (St. Louis, MO, USA). Fuming nitric acid, urea, pentaerythritol, sulphuric acid (H_2_SO_4_), chloroform, ammonium carbonate ((NH_4_)_2_CO_3_), potassium chloride (KCl), acetone, ethanol, dioxane, hydrazine hydrate, dichloromethane (DCM), tetrahydrofuran (THF), hexanol, silver nitrate (AgNO_3_), petroleum ether (PE) and ethyl acetate (EA) were received from Sinopharm Chemical Reagent Co. (Shanghai, China). Succinic anhydride, SA, GSH, triethylamine, dicyclohexylcarbodiimide (DCC), 4-dimethylaminopyridine (DMAP), diethyl ether, benzotriazole-1-yl-oxytripyrrolidinophosphonium hexafluorophosphate (PyBop), dimethyl sulfoxide (DMSO), tetramethylammonium hydroxide (TMAOH), nitric acid (HNO_3_), phosphotungstic acid, cyclohexane, N,N-diisopropylethylamine (DIPEA) and 3-aminobenzeneboronic acid (*m*-APBA) were purchased from Aladdin Reagent Co. (Shanghai, China). 1,2-Distearoyl-sn-glycero-3-phosphoethanolamine-N-(polyethylene glycol)-2000-carboxyl (DSPE-PEG2000-COOH) was obtained from ToYong Biotechnology Co. (Shanghai, China). 1-Hydroxybenzotriazole (HOBT) was acquired from J&K Chemical Ltd. (Beijing, China). Sepharose CL-2B columns and 4',6-diamidino-2-phenylindole (DAPI) were obtained from Solarbio Science & Technology Co., Ltd. (Beijing, China). Annexin V-FITC/PI Apoptosis Detection Kit was produced by KeyGEN Biotechnology Co., Ltd. (Nanjing, China). GSH Assay Kit, creatinine (CRE), blood urea nitrogen (BUN), alanine aminotransferase (ALT) and aspartate aminotransferase (AST) Detection Kits were purchased from Jiancheng Bioengineering Institute (Nanjing, China). Bicinchoninic acid (BCA) Assay Kit and 4% paraformaldehyde were obtained from Servicebio Technology Co., Ltd. (Wuhan, China). 1,2-Dioleoyl-sn-glycero-3-phosphate sodium salt (DOPA) was produced by Avanti Polar Lipids Inc (Alabaster, AL, USA). COS, 3-(4,5-dimethyl-thiazol-2-yl)-2,5-diphenyl tetrazolium bromide (MTT) and Triton X-100 were acquired from Macklin Biochemical Co., Ltd. (Shanghai, China). Nitric Oxide Assay Kit, Lyso-Tracker Red, 4-amino-5-methylamino-2′,7′-difluorofluorescein diacetate (DAF-FM DA) and radio immunoprecipitation assay (RIPA) lysis buffer were purchased from Beyotime Institute of Biotechnology (Shanghai, China). 1,2-distearoyl-sn-glycero-3-phosphoethanolamine-N-[methoxy(polyethylene glycol)-2000] (DSPE-mPEG2000) was supplied by AVT Pharmaceutical Tech Co., Ltd. (Shanghai, China). 1,1′-dioctadecyltetramethyl indotricarbocyanine iodide (DiR) was obtained from Absin Bioscience Inc (Shanghai, China). All reagents and solvents were of analytical or HPLC grade and used without further purification.

Fetal bovine serum (FBS) was obtained from Thermo Fisher Scientific (Chicago, IL, USA) and RPMI-1640 media was purchased from Procell Life Science & Technology Co., Ltd. (Wuhan, China). 4T1, MCF-7 and L02 cells were supplied by KeyGEN Biotechnology Co., Ltd. (Nanjing, China). In this study, cells from passage 5 to 15 were used to conduct experiments. 10% FBS and 1% penicillin–streptomycin were added to RPMI-1640 medium to prepare cell culture medium. Cells were cultured under hypoxic (1% O_2_) or normoxic (21% O_2_) conditions containing 5% CO_2_ at 37 ℃. The three-gas incubator was supplied by PUHE Biotechnology Co., Ltd. (Wuxi, China). When no special instructions were made on the experimental methods, the cells were cultured under hypoxic condition and the pH of culture medium was adjusted to 6.5.

BALB/c mice (female, 6–8 weeks) were purchased from Huafukang Bioscience Technology Co. (Beijing, China) and reared in the Experimental Animal Center of Huazhong University of Science and Technology.

### Synthesis and characterization of TPGS-3NO_3_ copolymer

The synthesis scheme of TPGS-3NO_3_ was illustrated in Additional file 1: Scheme S1.

### Synthesis of 3NO_3_-OH

Firstly, 3.5 mL fuming nitric acid was added to the round bottom flask and stirred for 20 min at -5 ℃. Subsequently, urea (7.5 mg, 0.125 mmol) and pentaerythritol (1.2 g, 8.81 mmol) were mixed evenly, and added to the flask in batches. The reaction solution was stirred for another 20 min. Then, 3.5 mL concentrated H_2_SO_4_ was added dropwise to the mixture, which was allowed to stir for 2 h at 0 ℃. After that, the above solution was slowly added dropwise to a vigorously stirred ice-water mixture (100 mL), and the precipitate was washed extensively with ultra-pure water and dried in vacuo overnight. Pentaerythritol tetranitrate is explosive, and hence we performed this reaction under low temperature with personal protection equipment.

Secondly, the crude product and ammonium carbonate (187.5 mg, 1.95 mmol) were dissolved in 60 mL acetone, and reacted for 30 min at 50 ℃. Then the mixture was added to 50 mL iced ethanol–water solution (36:64, v/v), and the precipitate was filtered and dried in vacuo overnight. Afterwards, the dried product was added to 90 mL ethanol/dioxane (1:1, v/v). Hydrazine hydrate (1.4 g, 28 mmol) was dissolved in 60 mL ultra-pure water and slowly dripped into the above solution which was allowed to reflux for 2.5 h. At the end of the reaction, the mixture was cooled, extracted with DCM, and the solvent was removed by rotary evaporation under vacuum. The resulting crude product was purified by silica gel column chromatography (eluent: PE/EA = 20/1, v/v).

The identity of product was determined by proton nuclear magnetic resonance spectroscopy (^1^H NMR) and carbon nuclear magnetic resonance spectroscopy (^13^C NMR) on a Bruker AV400 (Leipzig, Germany).

### Synthesis of TPGS-COOH

For the synthesis of TPGS-COOH, TPGS (1.51 g, 1 mmol), succinic anhydride (200.14 mg, 2 mmol), DMAP (122.17 mg, 1 mmol) and triethylamine (101.19 mg, 1 mmol) were stirred in 50 mL dioxane for 24 h at room temperature. Next, the mixture was evaporated to remove the solvent, dissolved in DCM, filtrated to remove the insoluble matter and concentrated to about 1 mL. Subsequently, the crude product was precipitated by pouring the 1 mL mixture into ice-cold diethyl ether, followed by removal of the solvent. After that, TPGS-COOH was added to the dialysis bag (MWCO 1 kDa), dialyzed against 20% ethanol for 24 h and finally collected by lyophilization.

### Synthesis of TPGS-3NO_3_

3NO_3_-OH (542.28 mg, 2 mmol), TPGS-COOH (1.6 g, 1 mmol), DCC (206.18 mg, 1 mmol) and DMAP (24.43 mg, 0.2 mmol) were dissolved in 20 mL anhydrous DCM and stirred at 40 ℃ for 24 h under nitrogen stream. After that, the precipitation was removed by filtration and DCM was removed by rotary evaporation under vacuum. The crude product was dissolved in a little DMSO and then purified by dialysis (MWCO 1 kDa) against ultra-pure water for 48 h. The product was finally collected by lyophilization.

### Characterization of TPGS-3NO_3_

The identity of product was determined by ^1^H NMR and high-resolution mass spectroscopy (HRMS) on a Bruker Daltonics SolariX 7.0 T (Leipzig, Germany).

### Synthesis and characterization of PBA-PEG2000-DSPE copolymer

PBA-PEG2000-DSPE was prepared by amide condensation, and its synthesis route was shown in Additional file 1: Scheme S2. Briefly, DSPE-PEG2000-COOH (233 mg, 0.08 mmol), PyBop (52.04 mg, 0.1 mmol), HOBT (15.31 mg, 0.1 mmol) and DIPEA (38.77 mg, 0.3 mmol) were dissolved in 15 mL anhydrous THF and stirred at room temperature for 4 h under nitrogen stream. Subsequently, *m*-APBA (13.69 mg, 0.1 mmol) was added to the mixture and stirred for 24 h. The mixed solution was then concentrated to 0.5 mL and added to 50 mL ice-cold diethyl ether. The precipitate was filtered, collected and dissolved in ultra-pure water to form micelles, which were purified by dialysis (MWCO 1 kDa) for 24 h to remove the by-products and unreacted impurities. The product was finally collected by lyophilization and then determined by ^1^H NMR and HRMS.

### Preparation and characterization of micelles

#### Synthesis of cisplatin prodrug *cis*- [Pt(NH_3_)_2_(H_2_O)_2_](NO_3_)_2_

*cis*- [Pt(NH_3_)_2_(H_2_O)_2_](NO_3_)_2_ was prepared by the method described previously. In brief, a mixture of 180 mg CDDP and 198.6 mg AgNO_3_ in 3 mL ultra-pure water was stirred at 60 ℃ for 3 h and then kept at room temperature overnight in the dark. Afterwards, the reaction solution was centrifuged at 10,000 rpm for 15 min to remove the AgCl precipitate and the supernatant was filtered using a 0.22 μm filter membrane.

### Preparation of cisplatin nanoparticles

Firstly, cyclohexane, Igepal CO-520, Triton X-100 and hexanol with a volume ratio of 288:87:15:10 were mixed to prepare the organic phase. Next, the 8 mL of above organic phase was added with 100 μL cisplatin prodrug and 149 μL DOPA (10 mg/mL), and the other 8 mL was added with 100 μL KCl solution (60 mg/mL), stirring for 20 min to form a uniform microemulsion, respectively. Then the two microemulsions were mixed, stirred for 30 min, demulsified with 16 mL ethanol and centrifuged for 15 min at 12,000 rpm. After washing with ethanol for 3 times, the precipitate was dissolved in 3 mL chloroform.

### Preparation of micelles

A mixture of TPGS-3NO_3_/PBA-PEG2000-DSPE (5 mg, 1:1, mol/mol) and 1 mL cisplatin nanoparticles were dispersed in 10 mL chloroform and then dried into a thin film by rotary evaporation under vacuum at 45 ℃. The lipid film was dispersed in 1 mL PBS to form TP/CDDP. Subsequently, 60 mg COS and a drop of TMAOH were added to TP/CDDP solution and incubated at 37 ℃ for 1 h. After purification by Sepharose CL-2B columns, CTP/CDDP micellar solution was obtained. The micelles without nitric oxide donors and PBA targeting ligands (denoted as D/CDDP), micelles without PBA targeting ligands and CDDP (denoted as TD, replaced PBA-PEG2000-DSPE with DSPE-mPEG2000), micelles without PBA targeting ligands (denoted as TD/CDDP) were also prepared using the same method, and their compositions were shown in Additional file 1: Table S1. In this study, micelles including TD, TD/CDDP, TP/CDDP, CTP/CDDP and D/CDDP were synthesized using cisplatin (denoted as CDDP), TPGS-3NO_3_ (denoted as T), PBA-PEG2000-DSPE (denoted as P), chitosan oligosaccharide (denoted as C) or DSPE-PEG2000-COOH (denoted as D).

### Characterization of micelles

The encapsulation efficiency of CDDP was measured by dissolving micelles in concentrated HNO_3_ and quantifying Pt concentration by SpectrAA-24OFS atomic absorption spectrometer (Varian, Palo Alto, CA, USA).

The average particle size and zeta potential of micelles were measured on a Zeta PALS Zeta Potential Analyzer from Brookhaven Instruments Corporation (Austin, TX, USA). The sample was negatively stained with 1% phosphotungstic acid, and the morphology and particle size of CTP/CDDP were observed under a JEOL 100CX II transmission electron microscope (TEM, Tokyo, Japan).

### In vitro stability

The stability of CTP/CDDP stored in refrigerator at 4 ℃ for 4 weeks was evaluated by taking samples every week and measuring the particle size and zeta potential at room temperature. To detect the leakage of drugs during storage, we sampled every 5 days. Free CDDP released from micelles was removed by size exclusion chromatography on Sepharose CL-2B columns and the residual CDDP was determined by atomic absorption spectrometer. For measuring the released NO, samples were detected by the Nitric Oxide Assay Kit.

### In vitro NO release

CTP/CDDP containing 400 μM NO was placed into the 20 mL glass bottle and incubated with or without 10 mmol/L GSH at 37℃ under mild stirring (150 rpm). At predetermined intervals from 0 to 72 h, an aliquot (50 μL) was taken out from the solution and detected by the Nitric Oxide Assay Kit.

### In vitro CDDP release

The release profile of CDDP from CTP/CDDP was studied using a dialysis method. 2 mL micellar solution was added to the dialysis bag (MWCO 10 kDa) and dialyzed against 400 mL PBS of different pH values (pH 7.4, 6.5, 5.5) at 37 ℃ under mild stirring (110 rpm). At selected time intervals from 0 to 72 h, an aliquot (50 μL) was taken from the dialysis bag and replaced with the same volume of fresh dialysate. The concentrations of CDDP in the samples were determined as described above.

### pH-sensitive affinity assay of PBA

PBS solutions of pH 6.5 and 7.4 were used to prepare a series of SA, COS and *m*-APBA solutions. The concentrations of COS and SA were 1, 2, 4, 8, 16, 32, 64 and 128 mmol/L, and the concentration of *m*-APBA was 120 μmol/L. SA (or COS) solution and *m*-APBA solution with the same pH value were mixed at 1:1 (v/v) and then incubated at 37 ℃ for 1 h. The fluorescence intensity of the mixed solution was determined by Hitachi F-4500 fluorescence spectrophotometer (Tokyo, Japan). The excitation wavelength was 302 nm and the emission wavelength was 388 nm. The combination stability assay of PBA-COS (*m*-APBA: 60 μmol/L, COS: 4 mM) was carried out using the same method. The mixture was incubated at 37 °C for 0.5, 1, 2, 4, 8, 12, 24, 36 and 48 h, respectively. After that, the fluorescence intensity was measured.

### In vitro cellular uptake

Cellular uptake of micelles in 4T1, MCF-7 and L02 cells was studied on an Olympus SZX12 fluorescence microscope (Tokyo, Japan). In the formation of micellar thin film, TD/CDDP/C6, TP/CDDP/C6 and CTP/CDDP/C6 were synthesized by adding fluorescent dye C6 to the mixture. The cells in logarithmic growth phase were plated in 12-well plates at a density of 10^5^ cells per well and the medium pH was adjusted to 7.4 or 6.5. After incubation overnight in a three-gas incubator, the cells were then treated with TD/CDDP/C6, TP/CDDP/C6, CTP/CDDP/C6 and CTP/CDDP/C6 + *m*-APBA (0.1 μg/mL C6 per well). Among these, in CTP/CDDP/C6 + *m*-APBA group, 1 mmol/L *m*-APBA was added to the cell culture medium 1 h before the administration of CTP/CDDP/C6. The cells were incubated for 3 h, washed with PBS for 3 times and fixed with 4% paraformaldehyde for 15 min. After that, the cells were washed with PBS for another 3 times, stained with DAPI (10 μg/mL) for 10 min and observed under a fluorescence microscope.

### Intracellular distribution of micelles

4T1 cells in logarithmic phase were seeded in confocal dishes (NEST Biotechnology, Wuxi, China) at a density of 10^5^ cells per well and incubated overnight in a three-gas incubator. CTP/CDDP/C6 containing 0.1 μg/mL C6 was then added to the media and co-incubated with 4T1 cells for 1 h or 3 h. Subsequently, the medium was abandoned and the Lyso-Tracker Red working solution (70 nM) preheated at 37 ℃ was added to the confocal dishes for an additional incubation of 2 h. Then the cells were washed with PBS for 3 times, fixed with 4% paraformaldehyde for 15 min, stained with DAPI (10 μg/mL) for 10 min and observed under a Nikon Eclipse Ti confocal laser scanning microscope (CLSM, Tokyo, Japan).

### Intracellular NO release

4T1 cells in logarithmic phase were plated in confocal dishes at a density of 10^5^ cells per well and incubated overnight. Then CTP/CDDP (8 μmol/L CDDP) was co-incubated with cells for 0 h, 12 h and 24 h, respectively. Afterwards, 1 mL diluted DAF-FM DA working solution (5 μmol/L) was added to the confocal dishes for an additional incubation of 20 min. Finally, the cells were washed with PBS for 3 times, fixed with 4% paraformaldehyde for 15 min, stained with DAPI (10 μg/mL) for 10 min, and then observed under CLSM.

### Cell cytotoxicity and apoptosis study

The proliferation inhibition of different drug formulations on 4T1 and MCF-7 cells under hypoxia and normoxia was evaluated by MTT method. In brief, the cells in logarithmic growth phase were seeded in 96-well plates at a density of 8 × 10^3^ cells per well and incubated overnight at 37 ℃. Cells were then incubated with serial dilutions of drug formulations for 24 h. After that, the medium was aspirated, and 20 μL freshly prepared MTT solution (5 mg/mL) was added and incubated for 4 h. The supernatants were removed carefully, followed by the addition of 150 μL DMSO per well. The purple crystal was completely dissolved by slight shaking, and the absorbance was measured on a Synergy H1 multiple detection microplate reader (BioTek, Winooski, VT, USA) at 490 nm. In addition, half-maximal inhibitory concentration (IC_50_) was used to evaluate the cytotoxicity of drugs.

The qualitative apoptosis of 4T1 cells treated with different drug formulations was detected by Annexin V-FITC/PI double staining. Briefly, 4T1 cells were plated in 12-well plates at a density of 2 × 10^5^ cells per well, followed by overnight incubation at 37 ℃. The cells were then treated with medium containing different drug formulations (20 μmol/L CDDP or 40 μmol/L NOD) for 24 h. Afterwards, the cells were washed with PBS for 3 times, trypsinized, centrifuged, and resuspended in 200 μL annexin V binding buffer. 5 μL Annexin V-FITC and 5 μL PI were then added and co-incubated with cells in the dark for 15 min. Finally, the cell samples were analyzed by a BD Accuri C6 flow cytometry (New York, NY, USA).

### Drug efflux and accumulation

4T1 cells (2 × 10^5^ cells per well) were plated in 12-well plates and incubated overnight at 37 ℃. Free CDDP, TD/CDDP, TP/CDDP and CTP/CDDP (20 μmol/L CDDP or 40 μmol/L NOD) were then added to the medium and incubated for 20 h. Subsequently, the cells were washed twice with PBS and RIPA lysis buffer was added. After centrifugation (12,000 rpm) at 4 ℃, the supernatant was collected. The concentrations of CDDP and protein were determined by a Prodigy Plus inductively coupled plasma optical emission spectrometer (ICP-OES, Leeman Labs, Mason, OH, USA) and BCA Assay Kit, respectively. To investigate the drug efflux and retention, 4T1 cells were treated with different drug formulations for 20 h, respectively. After washing twice with PBS, the cells were further incubated with fresh medium for another 4 h at 37 °C. Then, the cells were collected and washed with PBS to determine the CDDP retained in the cells using the same method.

### Intracellular GSH level

4T1 cells in logarithmic phase were seeded in 6-well plates at a density of 5 × 10^5^ cells per well and incubated overnight in a three-gas incubator. The cells were then treated with different drug formulations (20 μmol/L CDDP or 40 μmol/L NOD). After incubation of 24 h, the cells were washed twice with PBS and incubated with RIPA lysis buffer for 10 min. Next, the supernatant was collected after centrifugation at 12,000 rpm for 15 min. The concentrations of intracellular protein and GSH were determined by BCA Assay Kit and GSH Assay Kit, respectively.

### Wound healing assay

The ability of cell migration was evaluated by wound healing assay. 4T1 cells (5 × 10^5^ cells per well) in logarithmic phase were plated in 6-well plates and allowed to reach 100% confluence overnight. Subsequently, cell monolayers were wounded with a 200 μL pipette tip and the suspension cells were rinsed with PBS. The cells were then incubated with serum-free medium containing PBS or different drugs (20 μmol/L CDDP or 40 μmol/L NOD) for 24 h. Images of cells were taken at 0 and 24 h with a microscope. Finally, the width of scratches was measured and migration distance was calculated.

### Transwell migration and invasion assay

Transwell insert chambers (Corning, San Diego, CA, USA) with pore size of 8 μm and diameter of 6.5 mm were used for migration and invasion assay. For the migration assay, 5 × 10^4^ 4T1 cells were seeded in the inner chambers and incubated with serum-free medium containing PBS or different drugs (20 μmol/L CDDP or 40 μmol/L NOD). 600 μL medium containing 10% FBS was added to the lower chambers. After incubation of 24 h, the non-migratory cells on the upper surface of polycarbonate membrane were gently erased with cotton swabs. The migratory cells on the lower surface were fixed with 4% paraformaldehyde for 15 min, stained with 1% crystal violet solution, and finally observed by microscope. Similar to the migration experiment, the cell invasion test was also determined with transwells, and the only modification was that the surface of the polycarbonate membrane was pre-coated with a layer of Matrigel (Sigma-Aldrich) to simulate the extracellular matrix.

### Cellular immunofluorescence assay

4T1 cells in logarithmic phase were plated in 6-well plates at a density of 4 × 10^5^ cells per well and incubated overnight at 37 ℃. Then free CDDP, TD, TD/CDDP, TP/CDDP and CTP/CDDP (20 μmol/L CDDP or 40 μmol/L NOD) were added and co-incubated with cells for 24 h. Afterwards, the cells were washed with PBS for 3 times, fixed with 4% paraformaldehyde for 15 min and permeated with 0.1% Triton X-100 for 1 h. After incubation with E-cadherin (1:200, Cell Signaling, Danvers, MA, USA) or N-cadherin antibodies (1:200, Cell Signaling) overnight at 4 ℃, the cells were washed with TBST, incubated with Cy3-labeled goat anti-mouse IgG H&L antibody (1:200, Proteintech, Chicago, IL, USA) and observed by fluorescence microscope.

### Western blot

4T1 cells were seeded in 6-well plates and incubated overnight at 37 ℃. Different drug formulations were then added. After 24 h incubation, the cells were washed twice with PBS and incubated with RIPA lysis buffer for 10 min. The supernatant was collected after centrifugation (12,000 rpm) at 4 ℃ and the protein concentration was measured by BCA Assay Kit. After separation by gel (10% SDS-PAGE) electrophoresis, the proteins were electrotransferred to PVDF membranes, blocked with 5% skim milk for 1 h, and incubated with antibodies against β-tubulin (1:1000, Cell Signaling), MRP2 (1:1000, Cell Signaling), HIF-1α (1:1000, Cell Signaling), MMP9 (1:1000, Cell Signaling) and RKIP (1:1000, Cell Signaling) overnight at 4 ℃. PVDF membranes were then washed with TBST for 3 times and incubated with goat anti-rabbit IgG (H + L), HRP conjugated antibody (1:2000, Cell Signaling) for 1 h at room temperature. Finally, the expression of protein was detected by GeneGenome5 chemiluminescence system (Syngene, Cambridge, UK).

### Biodistribution study

The in vivo imaging and tissue distribution of tumor-bearing mice were studied by using near-infrared small animal imaging system (Pearl Trilogy, LI-COR, Lincoln, NE, USA). Briefly, 4T1 cells were subcutaneously injected in the right hindlimb of female BALB/c mice to establish xenograft mouse model. In the formation of micellar thin film, TD/CDDP/DiR, TP/CDDP/DiR and CTP/CDDP/DiR were synthesized by adding near-infrared fluorescent dye DiR to the mixture. For the in vivo imaging experiment, mice were randomly divided into two groups, and administrated of Free DiR or CTP/CDDP/DiR at a dose of 5 μg DiR per mouse by tail vein injection. The fluorescence intensity of DiR was scanned by near-infrared small animal imaging system at 3, 6, 12 and 24 h after administration. For the tissue distribution study, TD/CDDP/DiR, TP/CDDP/DiR and CTP/CDDP/DiR were injected into tumor-bearing mice through tail vein at a dose of 5 μg DiR per mouse. After administration of 12 h, mice were sacrificed and tissue samples including heart, liver, spleen, lung, kidney and tumor were collected. After washing with saline, tissues were scanned by a small animal imaging system. For the quantitative determination of CDDP concentrations in tissues, 4T1 xenograft tumor-bearing mice were given a single dose of TD/CDDP, TP/CDDP or CTP/CDDP (2 mg/kg) via tail vein, and then sacrificed at 12 h after injection. Hearts, livers, spleens, lungs, kidneys and tumor tissues were collected after sacrifice. The concentration of CDDP was determined by atomic absorption spectrometer as described above.

### Anti-tumor efficacy study in vivo

For the anti-tumor efficacy study, 10^6^ 4T1 cells in logarithmic growth phase were subcutaneously injected in the right hindlimb of female BALB/c mice. When tumor volume reached 100 mm^3^, mice were randomly divided into 6 groups (n = 5) and injected via the tail vein with PBS, free CDDP, TD, TD/CDDP, TP/CDDP and CTP/CDDP at a dose of 2 mg/kg CDDP on days 6, 8, 10 and 12 after tumor inoculation. Body weight and tumor size of mice were recorded every two days. Before sacrifice of mice, blood was collected via the retro-orbital and the plasma was separated by centrifugation for the measurement of biochemical indexes including ALT, AST, BUN and CRE. Tumors were harvested, weighed and processed for immunohistochemical analysis.

### In vivo efficacy of drugs against metastasis

To establish the metastasis model, 4T1 cells (5 × 10^5^) were injected into female BALB/c mice through tail vein. Then the mice were randomly divided into 6 groups (n = 5) and administrated of different drugs (2 mg/kg CDDP) on days 2, 4, 6 and 8 after tumor inoculation. Three weeks later, mice were sacrificed and lung tissues were excised. After washing with saline, the lungs were fixed with Bouin's solution and photographed. The pulmonary metastatic nodules were observed and counted under magnifying glass. After that, the lung tissues were embedded in paraffin and stained with hematoxylin and eosin (H&E).

### Statistical analysis

Data were presented as mean ± standard deviation (SD). Student's t-test and one-way ANOVA were performed for statistical analysis using SPSS Software (Chicago, IL, USA). Significant differences between groups were indicated by *p < 0.05, **p < 0.01 and ***p < 0.001.

## Results and discussion

### Synthesis and characterization of TPGS-3NO_3_ and PBA-PEG2000-DSPE

The synthesis scheme of TPGS-3NO_3_ was illustrated in Additional file 1: Scheme S1. ^1^H and ^13^C NMR results of 3NO_3_-OH were shown in Additional file 1: Figs. S1 and S2. ^1^H NMR (DMSO-d6): δ 5.35 (1H, OH), 4.62 (6H, NO_3_CH_2_), 3.51 (2H, OCH_2_) ppm. ^13^C NMR (DMSO-d6): δ 71.73 (NO_3_CH_2_), 59.70 (OCH_2_), 43.16 (C) ppm. TPGS-3NO_3_ was dissolved in chloroform-d and detected by ^1^H NMR. The result showed principal peaks (in ppm) assigned to the TPGS moiety (δ 3.61 ppm), the succinate moiety (δ 2.62 and 2.68 ppm), and the 3NO_3_ moiety (δ 4.24 ppm) (Additional file 1: Fig. S3). In addition, the result of HRMS indicated that the average molecular weight of TPGS-3NO_3_ was about 1,852 Da (Additional file 1: Fig. S4).

The PBA targeting ligand (PBA-PEG2000-DSPE) was prepared by amide condensation. The result of ^1^H NMR showed principal peaks (in ppm) assigned to the DSPE-PEG (δ 3.66 ppm) moiety and the *m*-APBA moiety (δ 7.32–7.77, 4.26 ppm) (Additional file 1: Fig. S5). As shown in Additional file 1: Fig. S6, the result of HRMS indicated that the average molecular weight of PBA-PEG2000-DSPE was about 3,003 Da.

### Preparation and characterization of micelles

**Fig. 2:**
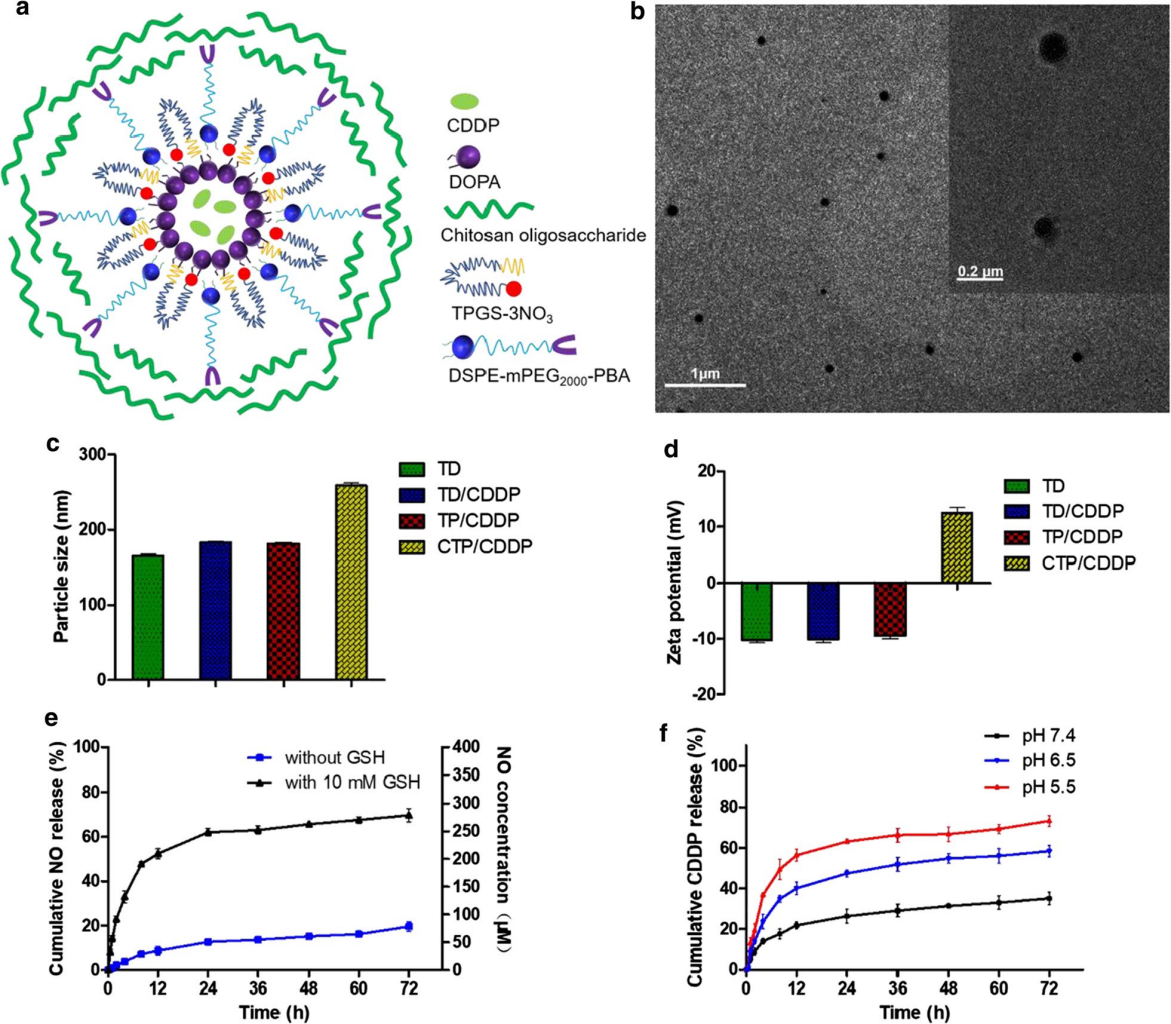
Characterization and drug release properties of micelles. **a** Schematic representation and **b** TEM image of CTP/CDDP. **c** Particle size and **d** zeta potential of TD, TD/CDDP, TP/CDDP and CTP/CDDP. **e** In vitro release of NO from CTP/CDDP incubated with or without 10 mmol/L GSH at 37 ℃. **f** In vitro release of CDDP from CTP/CDDP at pH 5.5, 6.5 and 7.4. Data are expressed as mean ± SD (n = 3)

The design of CTP/CDDP was illustrated in Fig. 2a. To improve solubility, CDDP was reacted with AgNO_3_ to form a water-soluble cisplatin prodrug, and the prodrug was further prepared into unilamellar lipid-coated cisplatin nanoparticles using a reverse microemulsion method. The nanoparticles were then loaded into the hydrophobic space inside the micelles through film dispersion method. Next, the COS shell on the surface of CTP/CDDP was conjugated with the PBA ligand via phenylboronate ester linkage. As shown in Fig. 2c, d, the particle size and zeta potential of micelles remained the same after CDDP loading. By contrast, COS modification increased the particle size from 181.02 nm to 258.64 nm, and reversed the zeta potential from negative to positive. The positive zeta potential might be attributed to the presence of the positively charged COS on the surface of CTP/CDDP. DLS measurements including intensity-, volume- and number-averaged particle size distributions indicated that micelles were uniform in size and well dispersed, with an average diameter of 258.64 nm and a PDI of 0.128 (Additional file 1: Fig. S7). The encapsulation efficiency of CDDP determined by atomic absorption spectrometry was 94.6 ± 3.7%. TEM analysis further confirmed the structure of micelles (Fig. 2b). The results showed that CTP/CDDP was spherical and the particle dimension was about 150 nm.

### In vitro stability and drug release properties

High stability is a prerequisite for the clinical research and application of nano-micelles. In this study, the stability of CTP/CDDP stored in refrigerator at 4 ℃ for 4 weeks was evaluated by observing the changes in particle size and zeta potential at room temperature. As shown in Additional file 1: Fig. S8, the average particle sizes at the beginning and the end of storage were 259.87 nm and 265.8 nm, respectively, and the zeta potentials were 12.40 mV and 12.13 mV, respectively. The particle size and zeta potential of CTP/CDDP did not change significantly within four weeks. More than 96% of drugs were still retained in CTP/CDDP after 20 days of storage (Additional file 1: Fig. S9). In addition, no micelle aggregation or drug precipitation was observed. These results collectively indicated the excellent stability of CTP/CDDP during storage.

It has been reported that nitrate esters can release NO under reductive conditions [36]. Hence, CTP/CDDP was co-incubated with 10 mmol/L GSH solution to investigate the responsive NO release in reductive microenvironment. The concentration of NO was determined using the Griess method with a UV spectrometer. Nitrite generated by oxidation of NO can react with Griess reagent under acidic conditions to produce an azo dye compound with a maximal absorbance at 540 nm. As revealed in Fig. 2e, the cumulative release rate of NO from CTP/CDDP at 72 h was less than 20% in PBS solution without GSH. However, an initial burst release of NO was observed in the presence of 10 mmol/L GSH. More than 200 μM (50%) NO was released from micelles within 12 h, and the cumulative release rate reached 70% at 72 h. Together, these results suggested that the release behavior of NO from CTP/CDDP was reduction-responsive.

Furthermore, the release profiles of CDDP from micelles were investigated by dynamic dialysis method. The microenvironment of normal blood, tumor extracellular matrix, and lysosomes was simulated using PBS buffer at pH 7.4, 6.5, and 5.5, respectively. As shown in Fig. 2f, after incubation at 37 ℃ for 72 h, 35.18% of encapsulated CDDP were released from CTP/CDDP in the buffer solution of pH 7.4 and 58.43% of CDDP was released at pH 6.5. However, at pH 5.5, the release rate of CDDP from micelles significantly increased, and the cumulative release percentage reached 73.28% at 72 h. CTP/CDDP exhibited pH triggered drug release, possibly due to the hydrolysis of phenylboronate ester bonds in micelles under acidic conditions. The deshielding of COS shell was able to accelerate the release of internally loaded drugs.

### In vitro cellular uptake

**Fig. 3:**
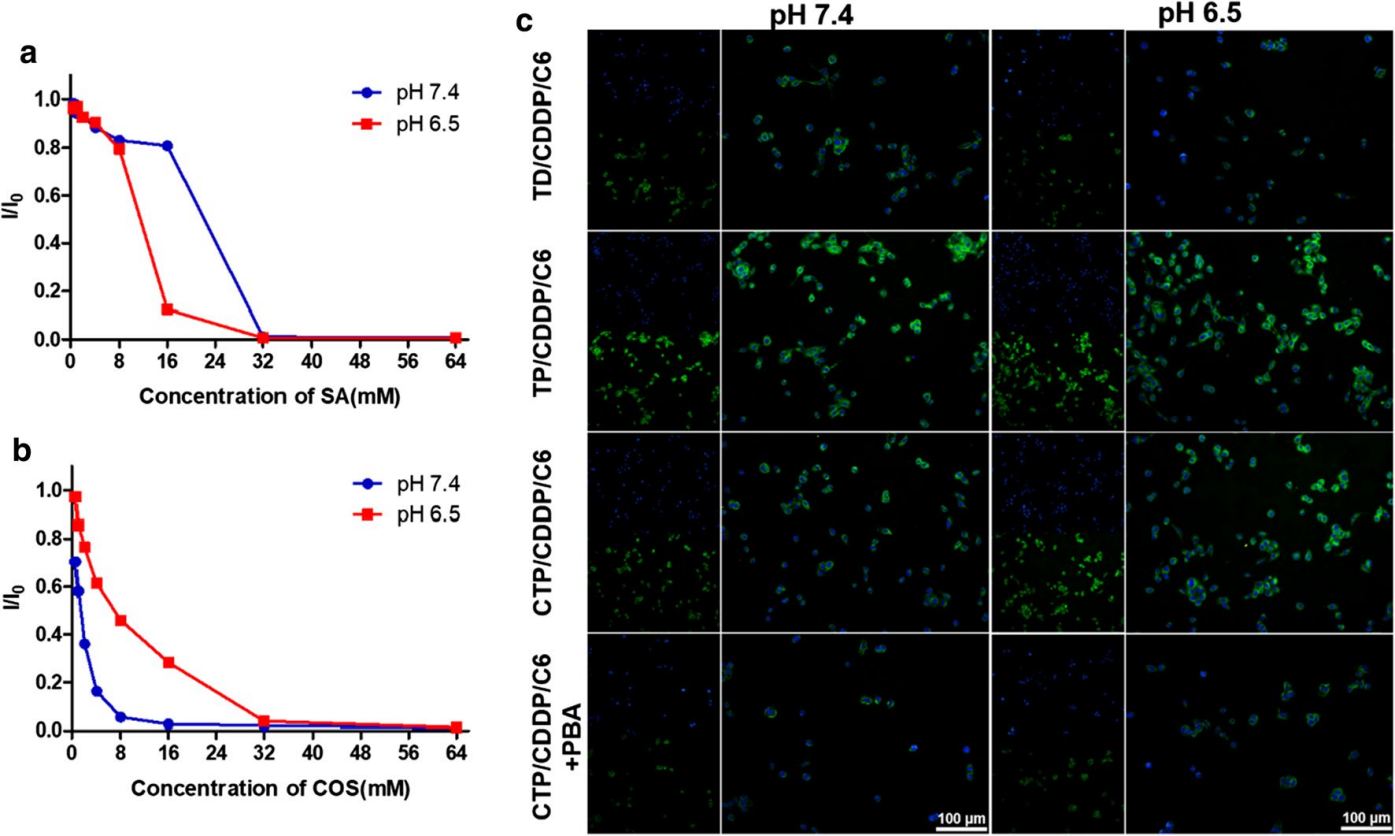
Cellular uptake of micelles in 4T1 cells. The affinity of free PBA to **a** SA or **b** COS in pH 7.4 or 6.5 PBS at 37 ℃ for 1 h. **c** Cellular uptake of 4T1 cells after incubation with different drug formulations at pH 6.5 or 7.4 for 3 h. Data are expressed as mean ± SD (n = 3)

Based on the different affinities of COS and SA to PBA under different pH conditions, an intelligent targeted nano-micelles were designed to enhance the cellular uptake of CDDP. Firstly, the in vitro binding characteristics of COS or SA with PBA were evaluated. *m*-APBA contains a fluorescent group with an excitation wavelength of 302 nm and an absorption wavelength of 388 nm [37]. When PBA combines with o-diol, the fluorescence would be quenched. Therefore, the affinities of COS and SA to PBA can be evaluated by the change of fluorescence intensity. As shown in Fig. 3a, b, the fluorescence intensity of *m*-APBA itself was scored as I_0_, and that after incubation with COS or SA was scored as I. According to the results of binding rate, the affinity of COS to PBA at pH 7.4 was stronger than that at pH 6.5. On the contrary, the affinity of SA to PBA at pH 6.5 was stronger than that at pH 7.4. The results of combination stability assay showed that reversible binding of PBA and COS was sensitive to the shift of pH (Additional file 1: Fig. S10). PBA-COS was prone to be dissociated at pH 6.5.

The cellular uptake efficiency of TD/CDDP/C6, TP/CDDP/C6 and CTP/CDDP/C6 at different pH were investigated in 4T1 and MCF-7 cells with high expression of SA residues. Fluorescent dye C6 was used to label the nano-micelles. The results showed that the uptake of those micelles in the two cancer cells were generally similar (Fig. 3c and Additional file 1: Fig. S11). Whether at pH 7.4 or 6.5, the uptake of TP/CDDP/C6 in 4T1 or MCF-7 cells was greater than that of TD/CDDP/C6, indicating that PBA ligands could increase the cellular uptake. Moreover, the cellular uptake of CTP/CDDP/C6 at pH 6.5 was greater than that at pH 7.4. A possible explanation was that CTP/CDDP/C6 could remove the shell of COS in a slightly acidic environment, leading to exposed PBA ligands, which combined with SA residues on the cell surface to increase drug uptake. These results suggested that PBA with high affinity to SA residues played a crucial role in cellular uptake as an accelerant. This hypothesis was further confirmed by the competitive inhibition assay, in which free *m*-APBA was able to compete with the micelles for SA residues, thereby reducing the internalization of CTP/CDDP/C6 (Fig. 3c and Additional file 1: Fig. S11).

SA residues are also expressed in some normal tissues, such as hepatocytes, so the uptake of micelles by L02 cells was further examined. As shown in Additional file 1: Fig. S12, the fluorescence intensity observed in L02 cells treated with TP/CDDP/C6 was higher than that with TD/CDDP/C6, suggesting that TP/CDDP/C6 was also able to enter cells via SA receptor-mediated endocytosis in normal hepatocytes. However, in the neutral environment of normal tissue at pH 7.4, the COS shell of CTP/CDDP/C6 could not be removed completely, hence there was no significant difference in cellular uptake between TD/CDDP/C6 and CTP/CDDP/C6.

Together, these results of drug uptake by 4T1, MCF-7 and L02 cells demonstrated that the COS shell was able to reduce the uptake of targeted micelles by normal cells through binding with PBA in neutral environment. By contrast, the outer layer of CTP/CDDP was deshielded in tumor acid environment, resulting in exposed PBA ligands, which were recognized by SA residues on the surface of tumor cells to enhance drug uptake.

Endocytosis is the main pathway for nanoparticles to enter tumor cells and the entrapment of endosomes/lysosomes is the major obstacle for CDDP entering cell nuclei. After co-incubation with 4T1 cells for 1 or 3 h, cells were stained with Lyso-Tracker Red and the intracellular distribution of CTP/CDDP/C6 was observed by CLSM. As shown in Additional file 1: Fig. S13, green fluorescence of CTP/CDDP/C6 could be observed in the lysosomes at 1 h, suggesting that CTP/CDDP/C6 entered cells and were trafficked to lysosomes. At 3 h, no green fluorescence of CTP/CDDP/C6 was observed in the lysosome, indicating that the CTP/CDDP/C6 had been digested and drugs had escaped from lysosomes after 3 h of incubation.

### Intracellular NO release

In vitro NO release experiment showed that CTP/CDDP could fully release NO under reductive conditions. Considering that the concentration of GSH in tumor cells was 1000 times higher than that in extracellular microenvironment, we speculated that CTP/CDDP could increase the intracellular NO level as well. DAF-FM DA is a fluorescent probe for the detection of NO, which can pass through the cell membrane by passive diffusion. After entering tumor cells, DAF-FM DA will be deacetylated by intracellular esterase to form DAF-FM that cannot pass through the cell membrane. DAF-FM itself has only weak fluorescence, but it can produce strong fluorescence after reacting with NO, so the intracellular NO concentration can be quantified by fluorescence intensity. As revealed in Additional file 1: Fig. S14, the appearance of weak green fluorescence at 0 h was attributed to the reaction between DAF-FM and endogenous NO. However, the intracellular fluorescence was significantly enhanced after the administration of CTP/CDDP and the concentration of NO in 4T1 cells at 24 h was higher than that at 12 h. The results indicated that CTP/CDDP, as a type of NOD, could release NO continuously and slowly in tumor cells.

### In vitro cytotoxicity and apoptosis

**Fig. 4:**
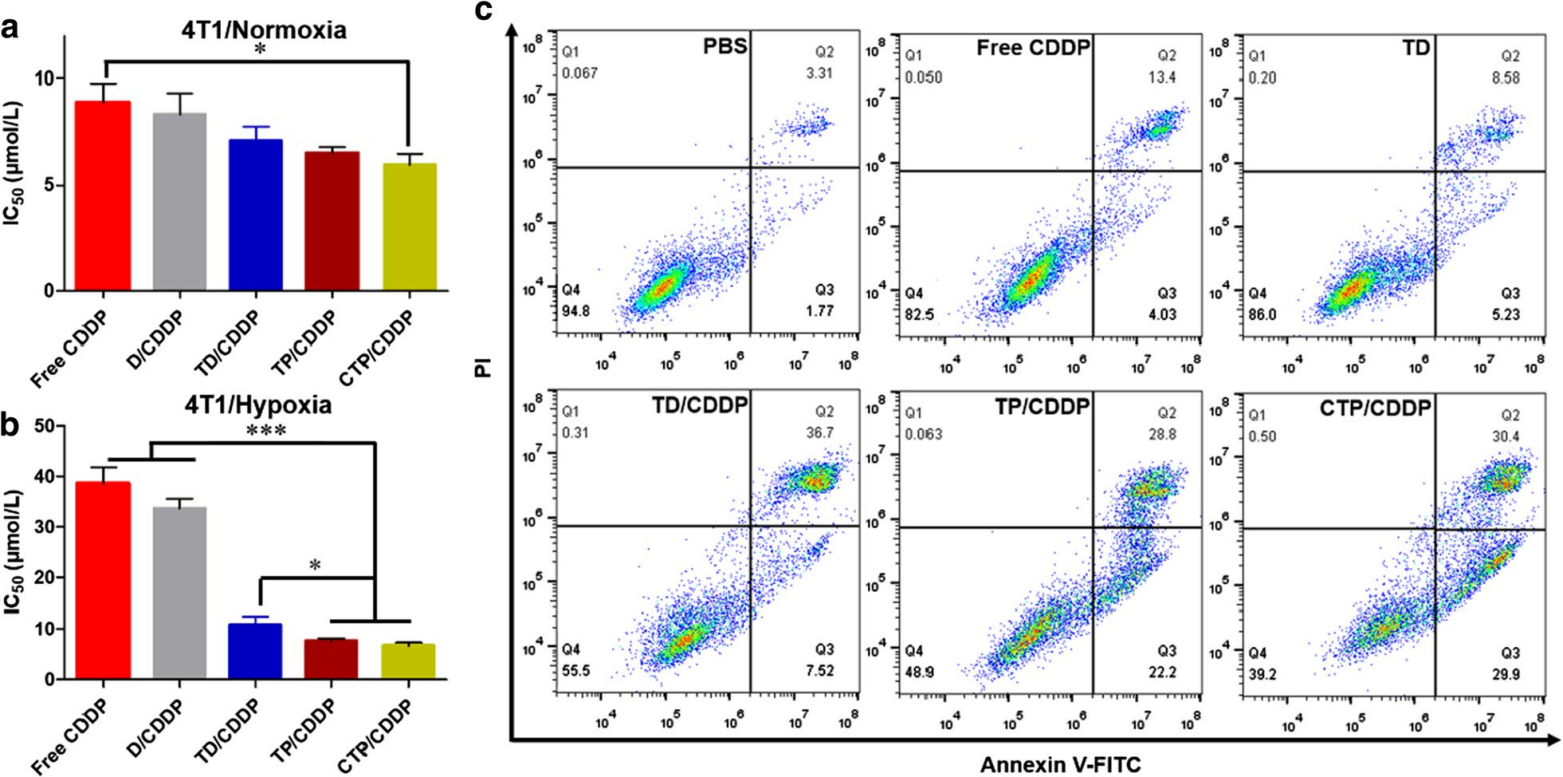
In vitro anti-tumor effects of different drug formulations in 4T1 cells. IC_50_ was calculated to evaluate the cytotoxicity of various drug formulations after incubation with 4T1 cells for 24 h under **a** normoxia and **b** hypoxia. **c** Apoptosis of 4T1 cells treated with different drug formulations for 24 h was detected by Annexin V-FITC/PI double staining. Cells treated with PBS were used as control. Data are expressed as mean ± SD (n = 3), *p < 0.05, ***p < 0.001

MTT assay was used to investigate the inhibitory effects of different drugs on the proliferation of 4T1 and MCF-7 cells under normoxic or hypoxic conditions. As shown in Fig. 4a, b, the IC_50_ values of free CDDP, D/CDDP, TD/CDDP, TP/CDDP and CTP/CDDP under normoxia were 8.89, 8.34, 7.10, 6.53 and 5.94 μmol/L, respectively. The results showed that the introduction of NOD and PBA ligands could enhance the anti-tumor effect of CDDP. Moreover, it has been reported that hypoxia contributes to the drug resistance of cancer cells, which leads to the decrease of CDDP efficacy [38]. A possible explanation for this phenomenon is the increase of GSH level and the high expression of efflux protein in hypoxic cancer cells. As expected, the IC_50_ of free CDDP under hypoxia was 4.3 times higher than that under normoxia, reaching 38.65 μmol/L. It was worth noting that the combination of CDDP and NOD could significantly improve the efficacy of chemotherapy drugs under hypoxic condition. The IC_50_ values of TD/CDDP, TP/CDDP and CTP/CDDP were 10.79, 7.67 and 6.72 μmol/L, respectively. The results showed that NO could reverse the drug resistance of 4T1 cells to CDDP. In addition, PBA-mediated endocytosis could further enhance the anti-tumor effect of micelles by increasing cellular uptake.

Similar phenomenon was observed in another breast cancer cell line, MCF-7. As shown in Additional file 1: Fig. S15, the IC_50_ of free CDDP under hypoxia was twice as much as that under normoxia, indicating that hypoxia could also induce resistance to CDDP in MCF-7 cells. Similarly, NO-releasing micelles could partially reverse MDR of hypoxic MCF-7 cells, and the IC_50_ values of TD/CDDP, TP/CDDP and CTP/CDDP were 25.74, 20.58 and 18.25 μmol/L, respectively. Under hypoxic condition, the effect of nano-micelles to overcome drug resistance in MCF-7 cells was weaker than that in 4T1 cells. Therefore, 4T1 cells were selected to further evaluate the anti-tumor effects of different drug formulations.

In order to further investigate the cytotoxicity of different drugs, Annexin V-FITC/PI double staining was performed to detect the apoptosis of hypoxic 4T1 cells. Due to MDR, free CDDP only caused apoptosis of 17.41% hypoxic 4T1 cells (Fig. 4c and Additional file 1: Fig. S16). In addition, the anti-tumor effect of TD alone is even worse than free CDDP. However, the combination of CDDP and NOD significantly enhanced the apoptotic effect. Induced apoptotic rates by TD/CDDP, TP/C DDP and CTP/CDDP were 43.16%, 51.47% and 56.13%, respectively. These results suggested that NOD enhanced the therapeutic effect of anti-tumor treatment mainly by reversing the drug resistance of cells to chemotherapeutic drugs. Consistent with the results of MTT, CTP/CDDP may further improved the cytotoxicity of CDDP to hypoxic 4T1 cells by increasing cellular uptake and releasing COS.

### Mechanism of reversing drug resistance

**Fig. 5:**
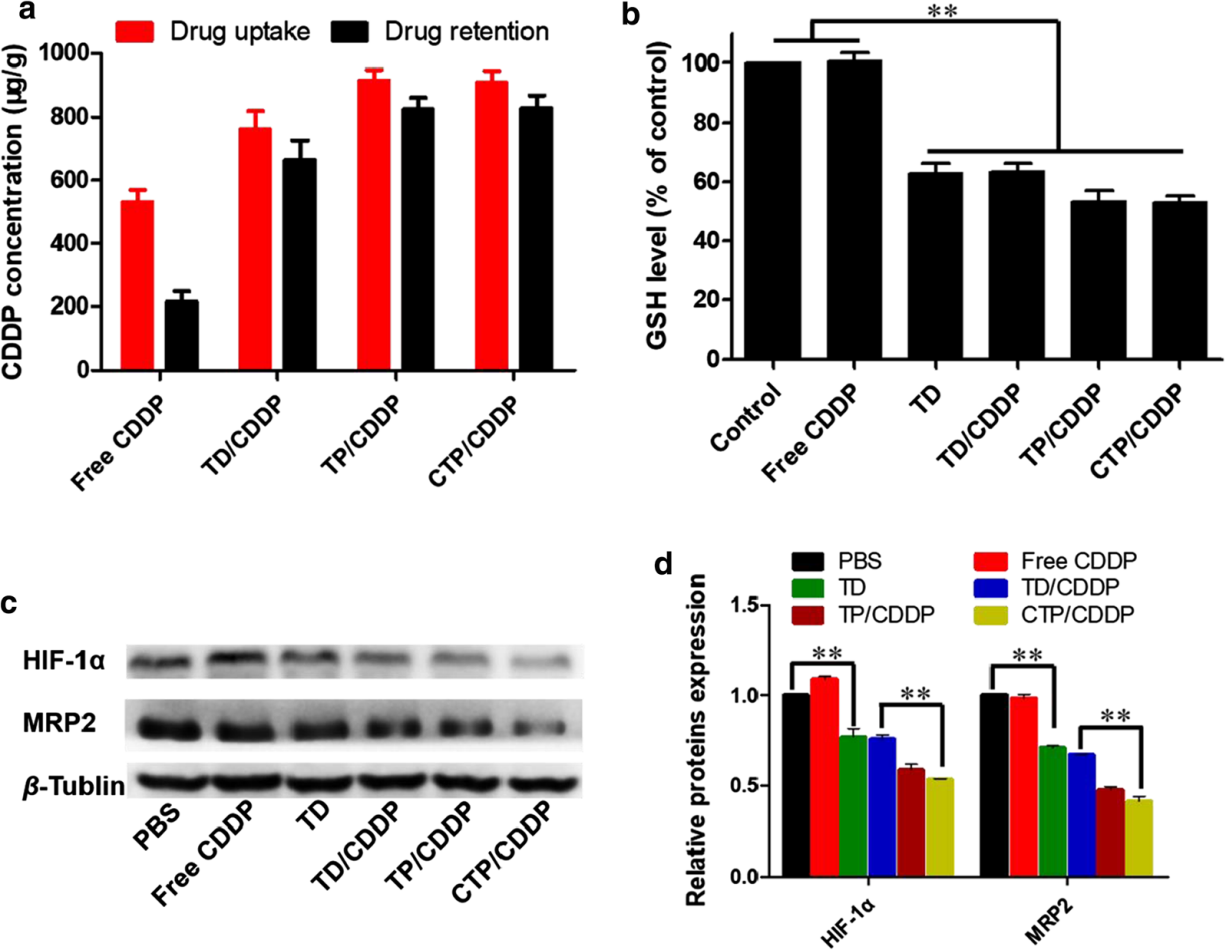
Mechanism of reversing drug resistance in vitro. **a** CDDP retention after incubation with free CDDP, TD/CDDP, TP/CDDP and CTP/CDDP for 20 h in hypoxic 4T1 cells. **b** Intracellular GSH levels of hypoxic 4T1 cells treated with various drug formulations for 24 h. **c** Western blot of indicated proteins expression in hypoxic 4T1 cells after 24 h incubation with different drugs. **d** The expression of relative proteins was calculated by the signal intensity of protein bands. Cells treated with PBS were used as control. Data are expressed as mean ± SD (n = 3), **p < 0.01, ***p < 0.001

In vitro cytotoxicity and apoptosis tests showed that NOD played an important role in reversing CDDP resistance. In order to explore the mechanism of NOD reversing MDR in hypoxic cancer cells, drug retention test was carried out firstly. As shown in Fig. 5a, the intracellular CDDP concentrations in TD/CDDP, TP/CDDP and CTP/CDDP administered groups were 3–4 times higher than that in free CDDP administered group, indicating that NOD could enhance the chemotherapeutic efficacy by increasing the cellular uptake and reducing the efflux of CDDP.

The classical pathway of CDDP efflux is that CDDP reacts with intracellular GSH to form a complex, which is then pumped out of cancer cell by MRP2. To verify whether NO could block this pathway to reduce CDDP efflux, we determined the GSH level and the expression of associated efflux protein after drug administration in hypoxic cells. As shown in Fig. 5b, the intracellular GSH concentrations were significantly decreased in the groups treated with NOD. It has been reported that NO released from nitrate esters requires the consumption of GSH and released NO can react with GSH to generate nitrosothiols [39]. This may be the main mechanism by which CTP/CDDP reduced the intracellular GSH level. In addition, our previous studies confirmed that hypoxia could induce HIF-1α and further enhance the expression of efflux protein, MRP2 [38]. As revealed in Fig. 5c, d, CTP/CDDP could down-regulate the expression of HIF-1α and MRP2. Taken together, it was speculated that CTP/CDDP was able to reduce the level of GSH and inhibit the HIF-1α/MRP2 pathway by releasing NO in hypoxic cells, thus reducing the efflux of CDDP and reversing the drug resistance of hypoxic cells.

### Synergetic anti-metastasis effect and mechanism

**Fig. 6:**
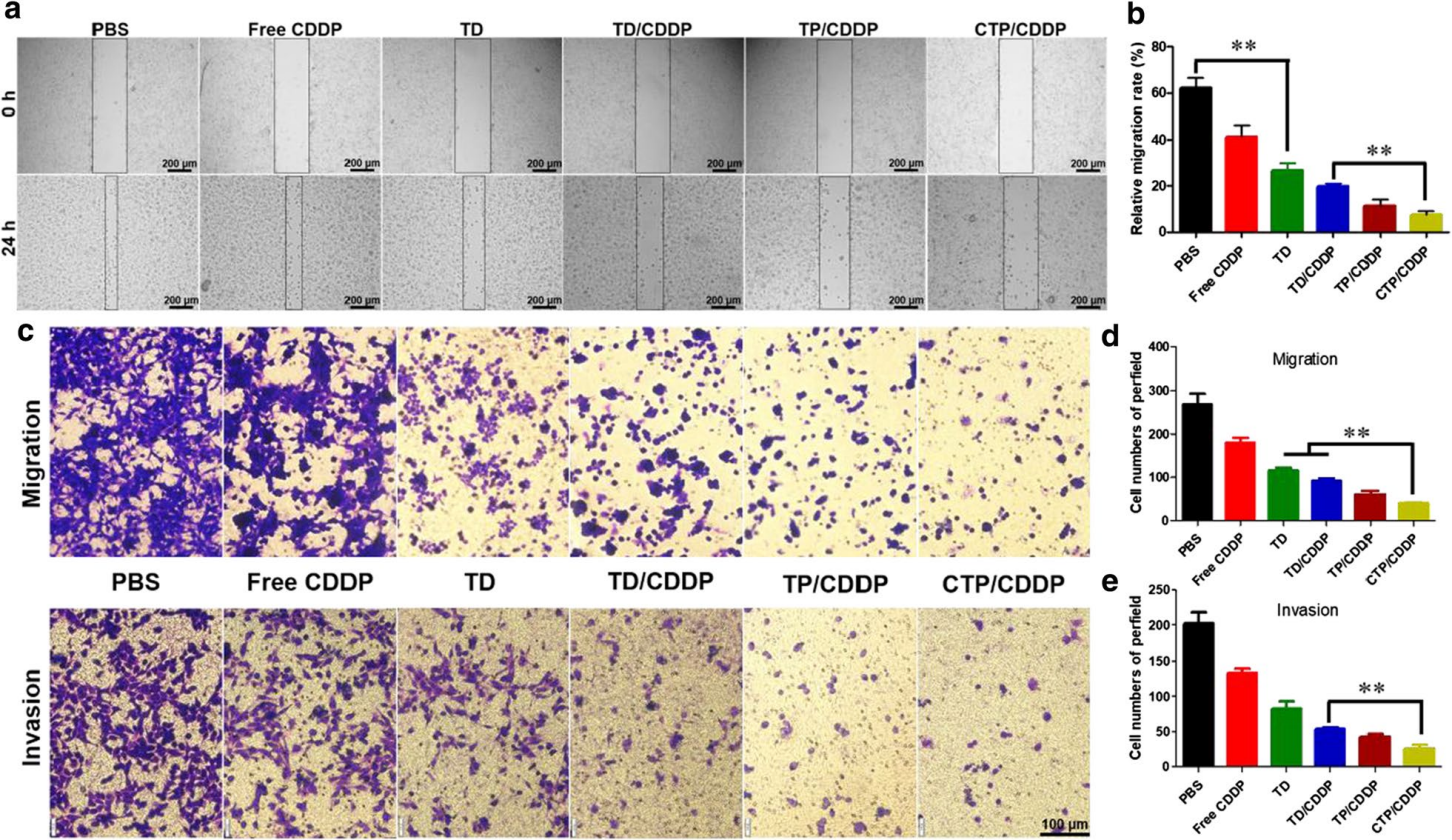
Synergetic anti-metastasis effect in vitro. **a** Images of 4T1 cells treated with PBS, free CDDP, TD, TD/CDDP, TP/CDDP and CTP/CDDP in wound healing assay at 0 and 24 h. **b** The quantification of wound healing assay was calculated as: percent closure (%) = length of cell migration/width of wounds × 100. Percent closure of control group was standardized as 100%. **c** Migration and invasion activities of 4T1 cells treated with various drug formulations for 24 h. The quantification of **d** migration and **e** invasion activities. Cells treated with PBS were used as control. Data are expressed as mean ± SD (n = 3), **p < 0.01

The hypoxic microenvironment of tumor not only leads to the chemotherapy resistance, but also promotes the metastasis of cancer cells. Therefore, the anti-metastasis effect of different drugs on tumor cells were evaluated. Firstly, the ability of cell migration after drug administration was examined by wound healing assay, and the results were shown in Fig. 6a, b. The scratch gap of the PBS group was significantly narrowed after 24 h incubation, and the cell migration rate reached 62.19%. However, the cell mobility decreased to 41.28% and 26.62% after the treatment of free CDDP and TD micelles, respectively. Furthermore, combined treatment of CDDP and NOD led to further reduced migration rates, which of TD/CDDP, TP/CDDP and CTP/CDDP groups were 19.69%, 11.28% and 7.49%, respectively.

The synergistic anti-metastasis effect of nano-micelles was further explored by transwell migration and invasion assay. As shown in Fig. 6c–e, the results of transwell migration and invasion were basically the same. The number of 4T1 cells that penetrated the polycarbonate membrane in the combined CDDP/NO groups were less than that in the free CDDP or TD treated groups, indicating a stronger inhibitory effect. Among them, CTP/CDDP showed the strongest ability to inhibit tumor cell metastasis, consistent with the results observed in wound healing assay. These results indicated that active targeting mediated by PBA ligands and COS enhanced the inhibitory effects on cancer cell metastasis.

**Fig. 7:**
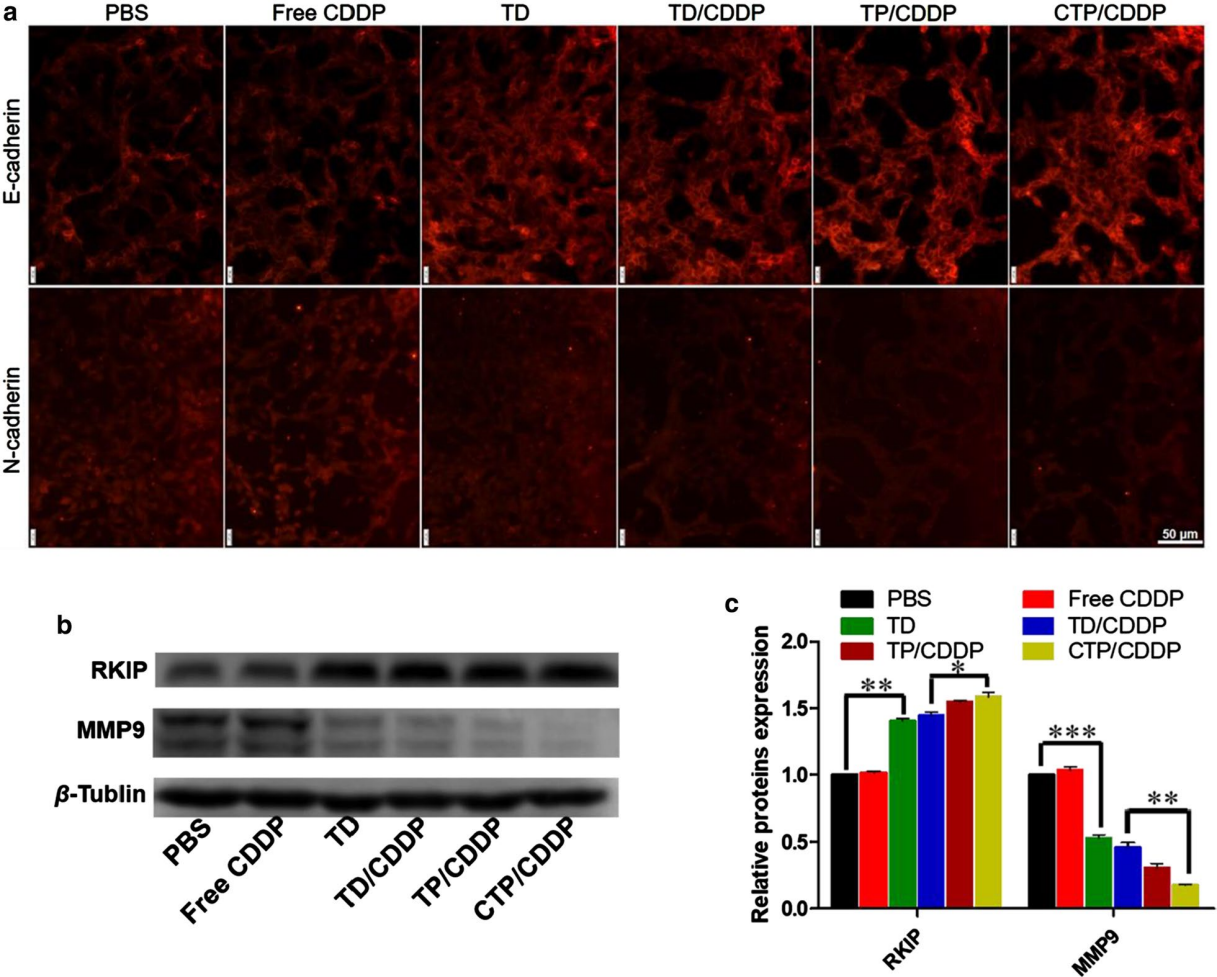
Mechanism of CTP/CDDP inhibiting tumor metastasis. **a** Immuno-fluorescence of EMT markers in 4T1 cells after administration with PBS, free CDDP, TD, TD/CDDP, TP/CDDP or CTP/CDDP for 24 h. **b** Western blot of indicated proteins expression in hypoxic 4T1 cells after 24 h incubation with different drugs. **c** The expression of relative proteins was calculated by the signal intensity of protein bands. Cells treated with PBS were used as control. Data are expressed as mean ± SD (n = 3), *p < 0.05, **p < 0.01, ***p < 0.001

NO can inhibit the EMT process, and subsequently reduce the metastatic activity of tumor cells [40, 41]. Therefore, the mechanism of CTP/CDDP inhibiting tumor metastasis was explored at the molecular level by detecting the expression of EMT markers and associated proteins. As shown in Fig. 7a, the EMT process of 4T1 cells was significantly inhibited after micellar treatment, as evidenced by the increased expression of E-cadherin and decreased expression of N-cadherin. Hypoxia can induce tumor cells to release MMP9, which is closely related to the process of EMT. As displayed in Fig. 7b, c, NO decreased the expression of MMP9 but enhanced the activity of RKIP. Moreover, the COS dissociated from CTP/CDDP further down-regulated the expression of MMP9. Taken together, CTP/CDDP potentiated RKIP expression and blocked the HIF-1α/MMP9 pathway by releasing NO and COS in hypoxic 4T1 cells, thereby inhibiting the EMT process and exerting a synergistic anti-metastatic effect.

### Biodistribution

**Fig. 8:**
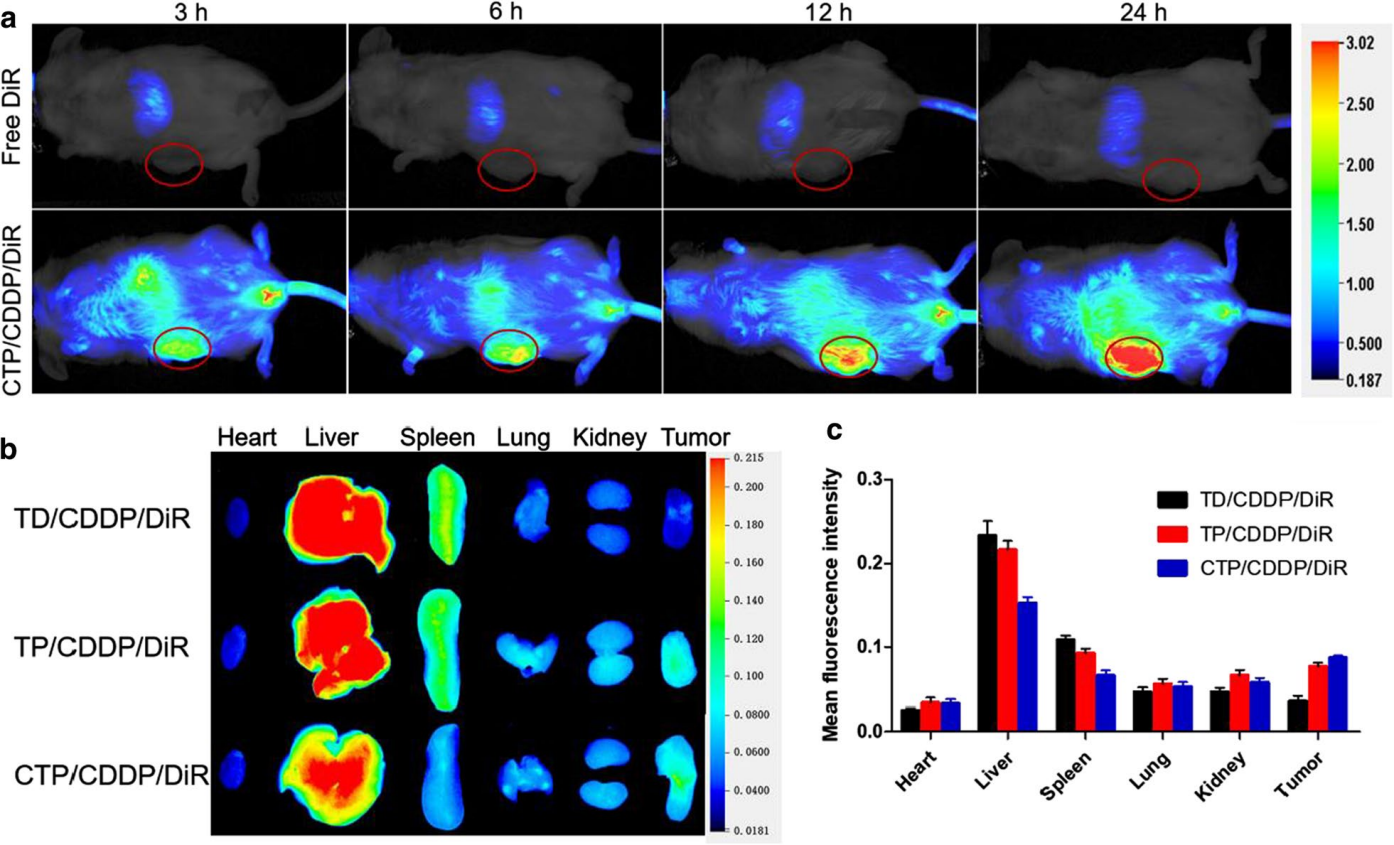
The in vivo imaging and tissue distribution of tumor-bearing mice. **a** In vivo images of 4T1 tumor-bearing mice administrated of free DiR or CTP/CDDP/DiR for 3 h, 6 h, 12 h and 24 h via tail vein injection. **b** Ex vivo images of organs and tumors excised from 4T1 xenograft tumor-bearing nude mice at 12 h after intravenous injection of TD/CDDP/DiR, TP/CDDP/DiR and CTP/CDDP/DiR. **c** Semiquantitative mean fluorescence intensity results of organs and tumors. Data are expressed as mean ± SD (n = 3)

First, we labeled micelles with near-infrared fluorescent dye DiR and then evaluated the tumor targeting efficacy of CTP/CDDP/DiR using in vivo imaging system. As shown in Fig. 8a, free DiR underwent rapid drug metabolism and blood circulation clearance after injection, and its fluorescence signal was barely detected within the tumor. By contrast, fluorescence could be observed clearly in the tumor tissues at 3 h after CTP/CDDP/DiR administration, and the fluorescence intensity increased over time course. The result confirmed that the nano-micelles possessed the preferable long circulation and tumor targeting capabilities.

The tissue distributions of different micelles were further compared by ex vivo imaging. As revealed in Fig. 8b, c, the tumor fluorescence of TP/CDDP/DiR group was higher than that of TD/CDDP/DiR group, suggesting that the binding of PBA ligands to SA residues increased uptake of micelles in tumor tissues. However, SA residues are also expressed on some normal tissues, resulting in increased uptake. In the design of this project, the outer layer COS and PBA ligands were covalently coupled via pH-sensitive phenylboronate ester linkage, which allowed CTP/CDDP to remain stable in the physiological microenvironment of normal tissues. Meanwhile, CTP/CDDP was able to remove the COS shell in the acidic microenvironment of tumor tissues, expose the PBA ligands and restore the affinity with SA residues. Therefore, compared with TP/CDDP/DiR group, the fluorescence intensity of CTP/CDDP/DiR group decreased in normal tissues, but increased in tumor tissues. This was confirmed by the biodistribution of CDDP in 4T1 xenograft mouse tissues at 12 h after the intravenous injection of TD/CDDP, TP/CDDP or CTP/CDDP (Additional file 1: Fig. S17). The results of biodistribution indicated that the intelligent targeted nano-micelles not only had active targeting ability to tumors, but also could reduce the uptake by normal tissue, which proved the feasibility of our design.

### In vivo antitumor activity and toxicity

**Fig. 9:**
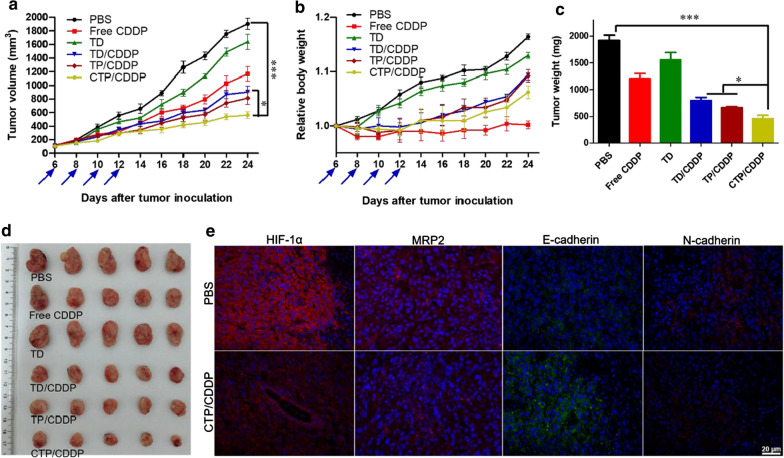
In vivo anti-tumor efficiency of different drug formulations in mice bearing 4T1 xenograft tumors. **a** Tumor growth curves and **b** relative body weight during treatment. Mice were injected via tail vein with PBS, free CDDP, TD, TD/CDDP, TP/CDDP or CTP/CDDP on days 6, 8, 10 and 12 after tumor inoculation. **c** Weight and **d** image of excised tumors at the end of the experiment (day 24 after tumor inoculation). **e** Immunohistochemical analysis including HIF-1α, MRP2, E-cadherin and N-cadherin of 4T1 xenograft tumors. Data are expressed as mean ± SD (n = 5), *p < 0.05, ***p < 0.001

The in vivo anti-tumor effect of CTP/CDDP was evaluated in 4T1 xenograft tumor-bearing mice. BALB/c mice were injected with different drugs via tail vein on days 6, 8, 10, and 12 after tumor cell inoculation. The relevant data, including tumor growth curves (Fig. 9a), weights (Fig. 9c) and picture (Fig. 9d) of dissected tumors were recorded to evaluate the anti-tumor effect. The results showed that the tumors of mice treated with PBS and TD grew rapidly and the average tumor weights reached 1916 and 1552 mg at the end of the experiment, respectively. Moreover, free CDDP showed a certain therapeutic effect in vivo, with an average volume of the final tumor at 1169.94 mm^3^. Since PBA ligands could improve the tumor targeting and aggregation of micelles, the average weight of tumors in TP/CDDP and CTP/CDDP groups decreased significantly. Compared with other drugs, CTP/CDDP showed the strongest antitumor effect, indicating that the intelligent targeting strategy enabled CTP/CDDP to successfully maintain the affinity for SA residues in the acidic TME. These results were further demonstrated by the immunofluorescence analysis, which showed the strongest TUNEL signal in tumor sections from the CTP/CDDP administrated mice group (Additional file 1: Fig. S18).

The changes of body weight and related biochemical indexes of liver and kidney in mice during treatment was determined to evaluate the systemic toxicity of different drugs. As shown in Fig. 9b, only the mice treated with free CDDP induced a significant weight loss. Similarly, free CDDP significantly increased the levels of BUN and CRE of mice, suggesting that CDDP could cause renal damage (Additional file 1: Fig. S19). However, no evident toxicity was observed in other administration groups, indicating low toxicity of CTP/CDDP.

To clarify the mechanisms of reversing drug resistance and anti-metastasis by nano-micelles, the above tumor sections were subjected to immunofluorescence staining, and the results were shown in Fig. 9e. Compared with free PBS group, the expression of HIF-1α, MRP2 and N-cadherin in CTP/CDDP group was significantly down-regulated, while the expression of E-cadherin was significantly up-regulated. The above immunohistochemical results were consistent with the results of in vitro experiments, indicating that NO could enhance the efficacy of CDDP and reduce cancer cell metastasis by inhibiting hypoxia-induced HIF-1 expression.

### In vivo anti-metastasis effect

**Fig. 10:**
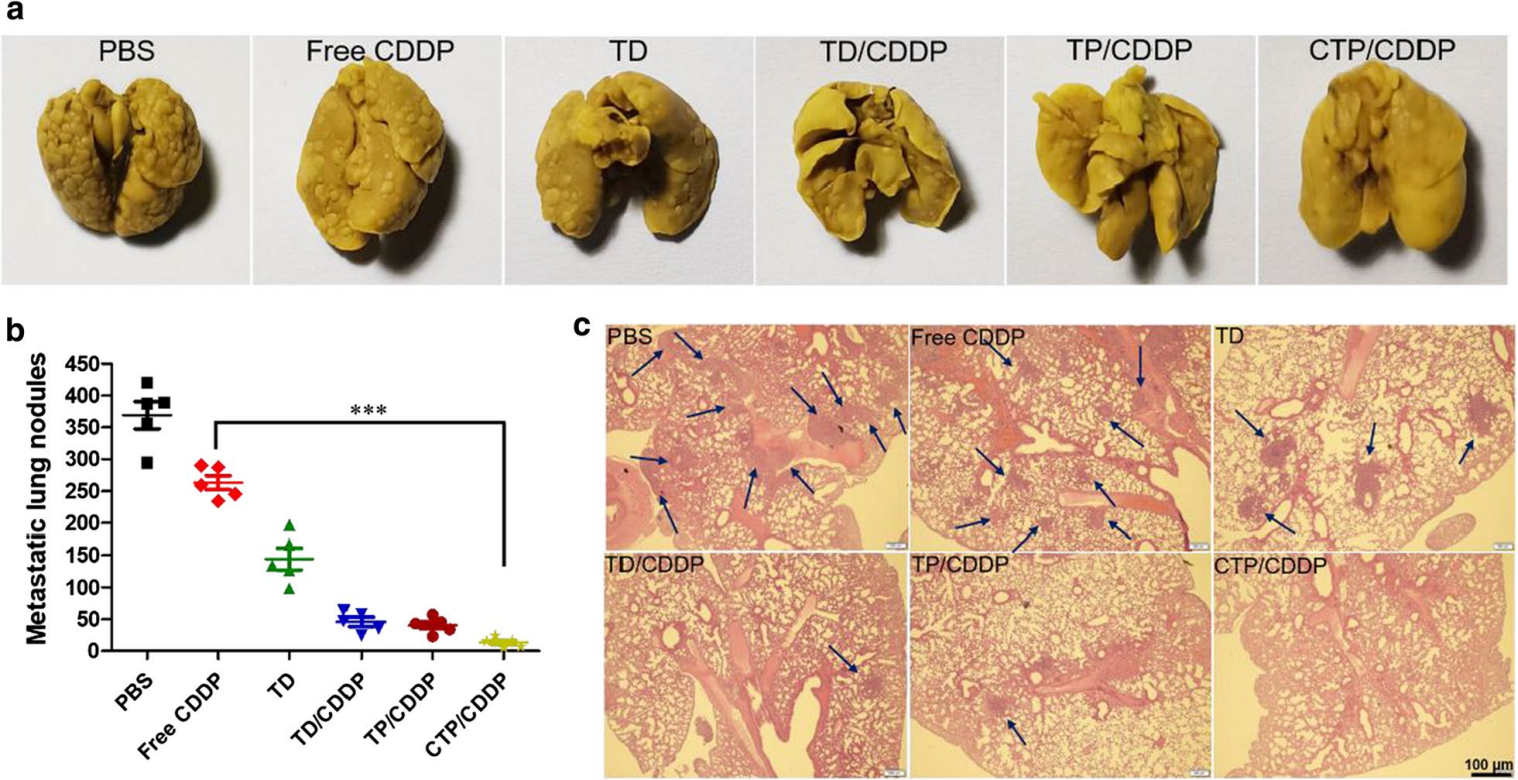
In vivo anti-metastasis effect. **a** Digital photographs of excised lung tissues at the end of the experiment (day 21 after tumor inoculation). Mice were injected with 4T1 cells through tail vein and administrated of PBS, free CDDP, TD, TD/CDDP, TP/CDDP or CTP/CDDP on days 2, 4, 6 and 8 after tumor inoculation. **b** Quantitative analysis of the metastatic lung nodules. **c** H&E staining of excised lung tissues. Data are expressed as mean ± SD (n = 5), ***p < 0.001

The immunofluorescence results of xenograft tumor sections showed that CTP/CDDP could effectively inhibit the EMT process. In order to intuitively evaluate the in vivo anti-metastasis effect, a lung metastasis model was established. At the end of the experiment, the lung tissues were dissected and fixed with Bouin's solution (Fig. 10a and Additional file 1: Fig. S20). The number of lung metastatic nodules was counted (Fig. 10b), and the lung tissues were examined under a microscope after H&E staining (Fig. 10c). Compared with the PBS group, the pulmonary metastatic nodules in TD, TD/CDDP, TP/CDDP, and CTP/CDDP groups were decreased by 60.89%, 87.65%, 99.85%, and 96.37%, respectively. In summary, intelligent targeted nano-micelles were suitable for the treatment of highly metastatic tumors by releasing NO and COS.

## Conclusions

In summary, COS-coated and SA receptor-targeted micelles were successfully prepared for co-delivery of CDDP and NO (CTP/CDDP). CTP/CDDP served as functions of both TME-responsive uptake and drug release. Cellular uptake and tissue distribution experiments demonstrated the intelligent targeting of this nano-micelles. Due to the different affinities of COS and SA to PBA under different pH conditions, CTP/CDDP was able to target tumor cells and increase cellular uptake while reducing toxicity to normal tissues. In addition, the in vitro release assay revealed that CTP/CDDP could rapidly release encapsulated CDDP and NO under the dual triggering of pH and GSH. Therefore, CTP/CDDP exhibited enhanced anti-tumor activity.

In terms of anti-cancer mechanism, CTP/CDDP was able to decrease efflux and increase intracellular retention of CDDP by reducing the expression of HIF-1α, GSH and MRP2, thus relieving the chemotherapeutic drug resistance. In addition, CTP/CDDP could block HIF-1α/MMP9 pathway and promote RKIP expression in hypoxic cancer cells, thus inhibiting the EMT process and enhancing the anti-metastatic efficacy. Therefore, CTP/CDDP represents a promising approach to treat resistance and metastatic tumors.

## Supplementary Information

**Additional file 1: Table S1.** Composition of different micelles. **Scheme S1.** Synthesis scheme of TPGS-3NO_3_. **Scheme S2.** Synthesis scheme of PBA-PEG2000-DSPE. **Fig. S1.** ^1^H NMR of 3NO_3_-OH in DMSO-d6. **Fig. S2.** ^13^C NMR of 3NO_3_-OH in DMSO-d6. **Fig. S3.** ^1^H NMR of TPGS-3NO_3_ in chloroform-d. Characteristic peaks as marked in the graphs. **Fig. S4.** HRMS of TPGS-3NO_3_. **Fig. S5.** ^1^H NMR of PBA-PEG2000-DSPE in chloroform-d. Characteristic peaks as marked in the graphs. **Fig. S6.** HRMS of PBA-PEG2000-DSPE. **Fig. S7.** Dynamic light scattering (DLS) size measurement of CTP/CDDP. **Fig. S8.** Stability of CTP/CDDP at 4 ℃ for 4 weeks as measured by the particle size and zeta potential during storage. Data are expressed as mean ± SD (n = 3). **Fig. S9.** Leakage rate of **a** NO and **b** CDDP from CTP/CDDP during storage at 4 ℃. Data are expressed as mean ± SD (n = 3). **Fig. S10.** The combination stability assay of PBA-COS conjugate at different time points. **Fig. S11.** Cellular uptake of MCF-7 cells after incubation with different drug formulations at pH 6.5 or 7.4 for 3 h. **Fig. S12.** Cellular uptake of L02 cells after incubation with different drug formulations at pH 7.4 for 3 h. **Fig. S13.** CLSM images of 4T1 cells after incubation with CTP/CDDP/C6 for 1 h or 3 h. **Fig. S14.** Intracellular NO level in 4T1 cells after administration of CTP/CDDP for 0, 12 and 24 h. **Fig. S15.** In vitro anti-tumor effects of different drug formulations. IC_50_ was calculated to evaluate the cytotoxicity of various drug formulations after incubation with MCF-7 cells for 24 h under **a** normoxia and **b** hypoxia. Data are expressed as mean ± SD (n = 3), *p < 0.05, **p < 0.01. **Fig. S16.** The quantification of cell apoptosis. Data are expressed as mean ± SD (n = 3), ***p < 0.001. **Fig. S17.** CDDP biodistribution in tissues of 4T1 xenograft tumor-bearing mice at 12 h after intravenous injection. Data are expressed as mean ± SD (n = 5). **Fig. S18.** In vivo anti-tumor efficiency of different drug formulations in mice bearing 4T1 xenograft tumors. Tumor apoptosis was determined by TUNEL assay. **Fig. S19.** Values of biochemical indexes of liver and kidney containing **(a)** BUN, **b** CRE, **c** ALT and **d** AST. Data are expressed as mean ± SD (n = 5), *p < 0.05, **p < 0.01. **Fig. S20.** In vivo anti-metastasis effect. Image of excised lung tissues at the end of the experiment (day 21 after tumor inoculation). Data are expressed as mean ± SD (n = 5).

## Data Availability

All data generated or analyzed during this study are included in this manuscript.

## Abbreviations

TME: Tumor microenvironment
COS: Chitosan oligosaccharide
SA: Sialic acid
CDDP: Cisplatin
NO: Nitric oxide
PBA: Phenylboronic acid
EMT: Epithelial-mesenchymal transition
MDR: Multidrug resistance
HIFs: Hypoxia inducible factors
ROS: Reactive oxygen species
ABC: ATP-binding cassette
P-gp: P-glycoprotein
GSH: Glutathione
MRP: Multidrug resistance-associated protein
MMPs: Matrix metalloproteinases
ECM: Extracellular matrix
RES: Reticuloendothelial system
TPGS: D-α-tocopherol polyethylene glycol 1000 succinate
C6: Coumarin 6
DCM: Dichloromethane
THF: Tetrahydrofuran
PE: Petroleum ether
EA: Ethyl acetate
DCC: Dicyclohexylcarbodiimide
DMAP: 4-Dimethylaminopyridine
PyBop: Benzotriazole-1-yl-oxytripyrrolidinophosphonium hexafluorophosphate
DMSO: Dimethyl sulfoxide
TMAOH: Tetramethylammonium hydroxide
DIPEA: N,N-diisopropylethylamine
m-APBA: 3-Aminobenzeneboronic acid
DSPE-PEG2000-COOH: 1,2-Distearoyl-sn-glycero-3-phosphoethanolamine-N-(polyethylene glycol:-2000-carboxyl
FBS: Fetal bovine serum
IC_50_: Half-maximal inhibitory concentration
ICP-OES: Inductively coupled plasma optical emission spectrometer
HOBT: 1-Hydroxybenzotriazole
DAPI: 4',6-Diamidino-2-phenylindole
CRE: Creatinine
BUN: Blood urea nitrogen
ALT: Alanine aminotransferase
AST: Aspartate aminotransferase
BCA: Bicinchoninic acid
DOPA: 1,2-Dioleoyl-sn-glycero-3-phosphate sodium salt
MTT: 3-(4,5-Dimethyl-thiazol-2-yl:-2,5-diphenyl tetrazolium bromide
DSPE-mPEG2000: 1,2-Distearoyl-sn-glycero-3-phosphoethanolamine-N-[methoxy(polyethylene glycol:-2000]
DiR: 1,1′-Dioctadecyltetramethyl indotricarbocyanine iodide
DAF-FM DA: 4-Amino-5-methylamino-2′,7′-difluorofluorescein diacetate
