# Supplementary material for: Nitric oxide-releasing micelles with intelligent targeting for enhanced anti-tumor effect of cisplatin in hypoxia
Source: J Nanobiotechnology. 2021 Aug 16;19:246. doi: 10.1186/s12951-021-00989-z (PMC8365946; doi:10.1186/s12951-021-00989-z)
Supplement: Supplementary file 1 — Additional file 1: Table S1. Composition of different micelles. Scheme S1. Synthesis scheme of TPGS-3NO3. Scheme S2. Synthesis scheme of PBA-PEG2000-DSPE. Fig. S1. 1H NMR of 3NO3-OH in DMSO-d6. Fig. S2. 13C NMR of 3NO3-OH in DMSO-d6. Fig. S3. 1H NMR of TPGS-3NO3 in chloroform-d. Characteristic peaks as marked in the graphs. Fig. S4. HRMS of TPGS-3NO3. Fig. S5. 1H NMR of PBA-PEG2000-DSPE in chloroform-d. Characteristic peaks as marked in the graphs. Fig. S6. HRMS of PBA-PEG2000-DSPE. Fig. S7. Dynamic light scattering (DLS) size measurement of CTP/CDDP. Fig. S8. Stability of CTP/CDDP at 4 ℃ for 4 weeks as measured by the particle size and zeta potential during storage. Data are expressed as mean ± SD (n = 3). Fig. S9. Leakage rate of a NO and b CDDP from CTP/CDDP during storage at 4 ℃. Data are expressed as mean ± SD (n = 3). Fig. S10. The combination stability assay of PBA-COS conjugate at different time points. Fig. S11. Cellular uptake of MCF-7 cells after incubation with different drug formulations at pH 6.5 or 7.4 for 3 h. Fig. S12. Cellular uptake of L02 cells after incubation with different drug formulations at pH 7.4 for 3 h. Fig. S13. CLSM images of 4T1 cells after incubation with CTP/CDDP/C6 for 1 h or 3 h. Fig. S14. Intracellular NO level in 4T1 cells after administration of CTP/CDDP for 0, 12 and 24 h. Fig. S15. In vitro anti-tumor effects of different drug formulations. IC50 was calculated to evaluate the cytotoxicity of various drug formulations after incubation with MCF-7 cells for 24 h under a normoxia and b hypoxia. Data are expressed as mean ± SD (n = 3), *p < 0.05, **p < 0.01. Fig. S16. The quantification of cell apoptosis. Data are expressed as mean ± SD (n = 3), ***p < 0.001. Fig. S17. CDDP biodistribution in tissues of 4T1 xenograft tumor-bearing mice at 12 h after intravenous injection. Data are expressed as mean ± SD (n = 5). Fig. S18. In vivo anti-tumor efficiency of different drug formulations in mice bearing 4T1 xenograft tumors. Tumor apopt [file 12951_2021_989_MOESM1_ESM.docx]

**Supplementary Tables**

**Table S1.** Composition of different micelles

|  | CDDP | TPGS-3NO_3_ | PBA-PEG2000-DSPE | DSPE-mPEG2000 | COS |
| --- | --- | --- | --- | --- | --- |
| TD |  | **√** |  | **√** |  |
| TD/CDDP | **√** | **√** |  | **√** |  |
| TP/CDDP | **√** | **√** | **√** |  |  |
| CTP/CDDP | **√** | **√** | **√** |  | **√** |
| D/CDDP | **√** |  |  | **√** |  |

**Supplementary Figures**


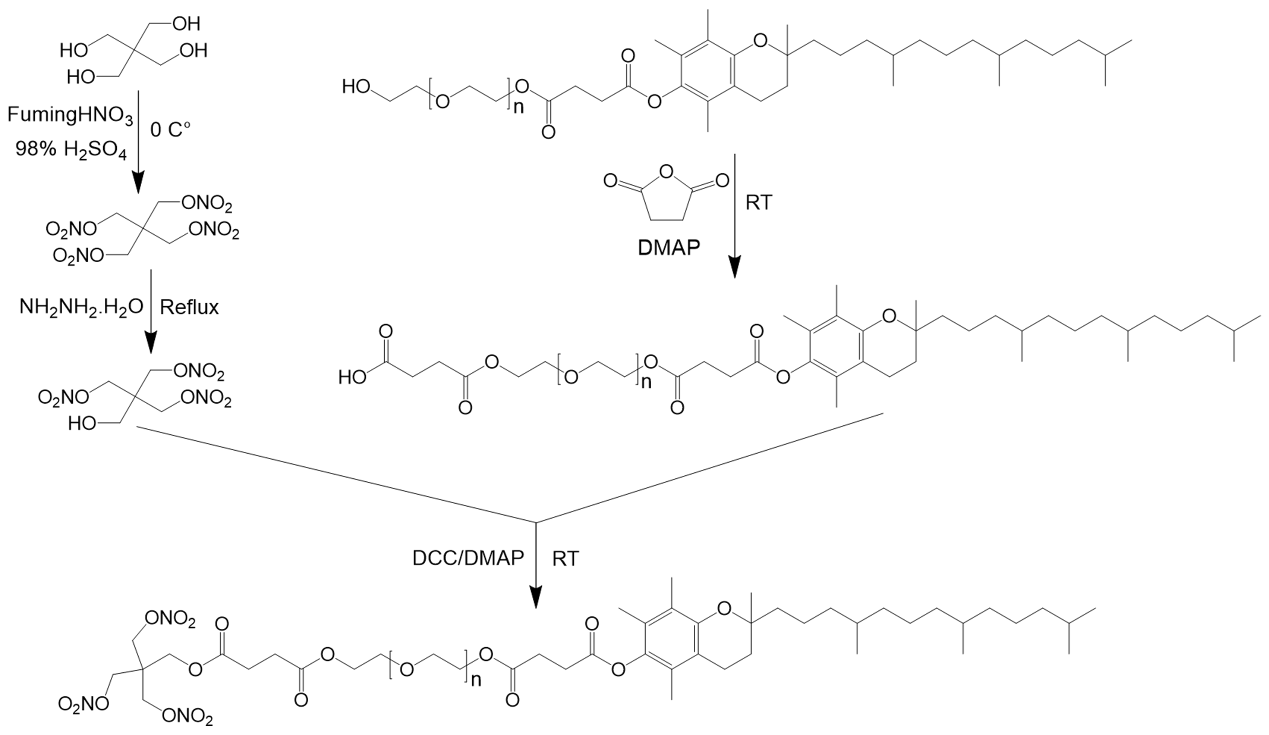


**Scheme S1.** Synthesis scheme of TPGS-3NO_3_.


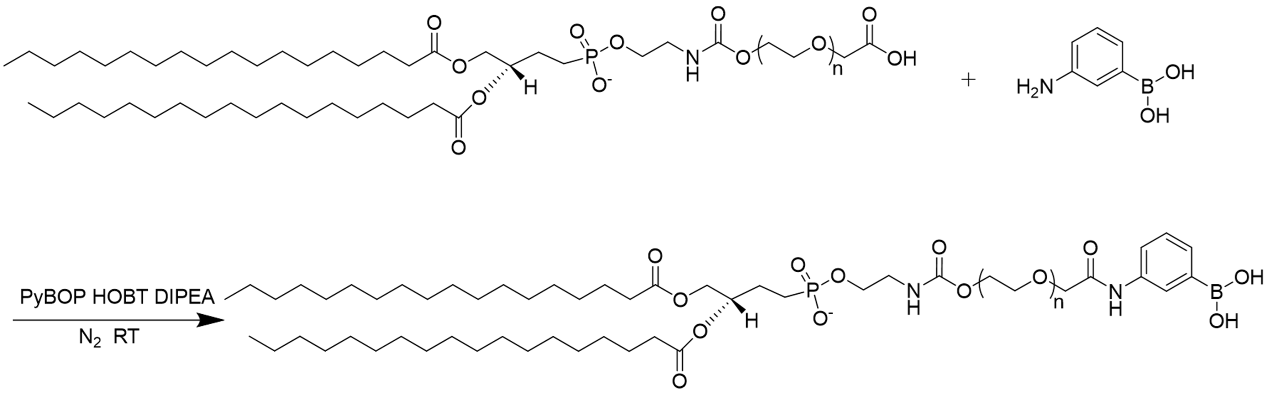


**Scheme S2.** Synthesis scheme of PBA-PEG2000-DSPE.

**
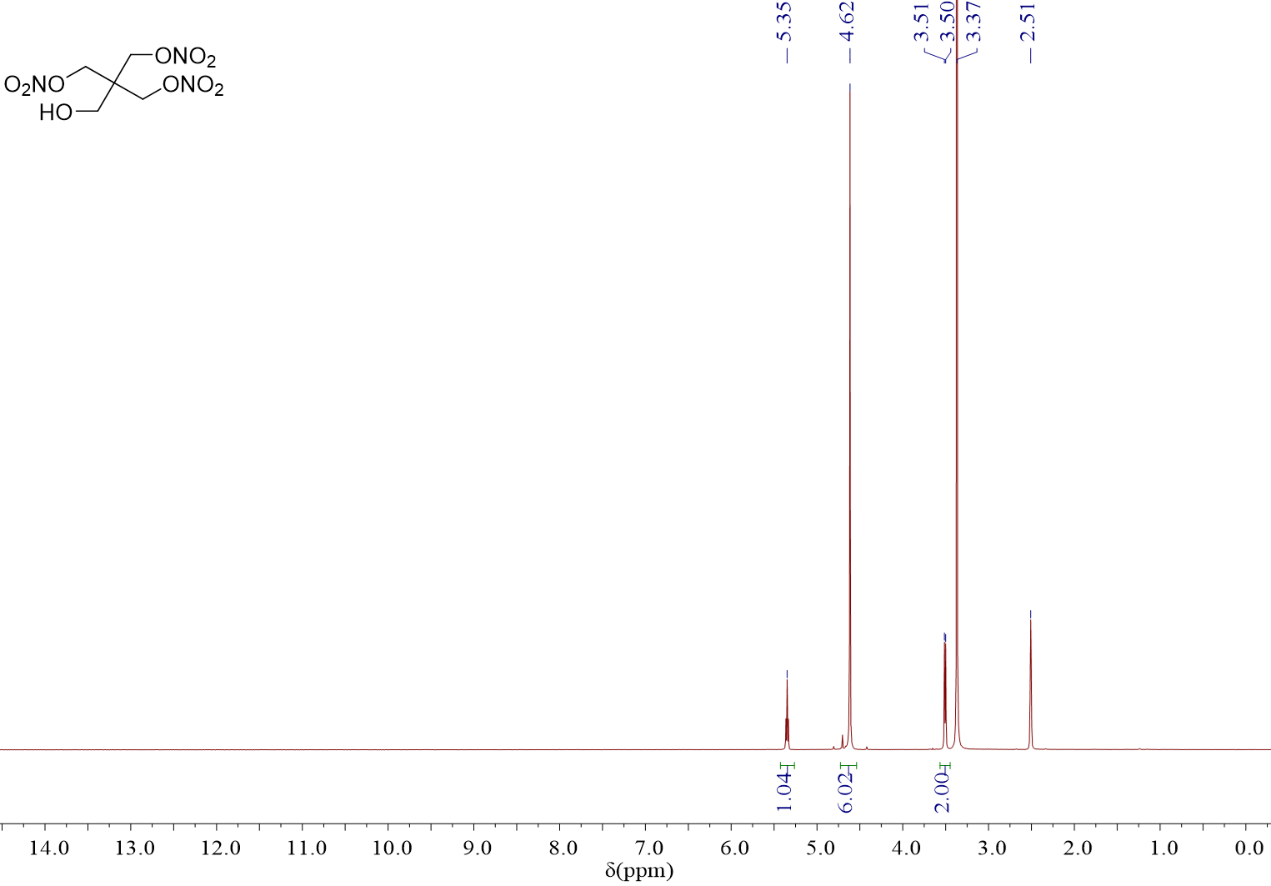
**

**Fig. S1.** ^1^H NMR of 3NO_3_-OH in DMSO-d6.

**
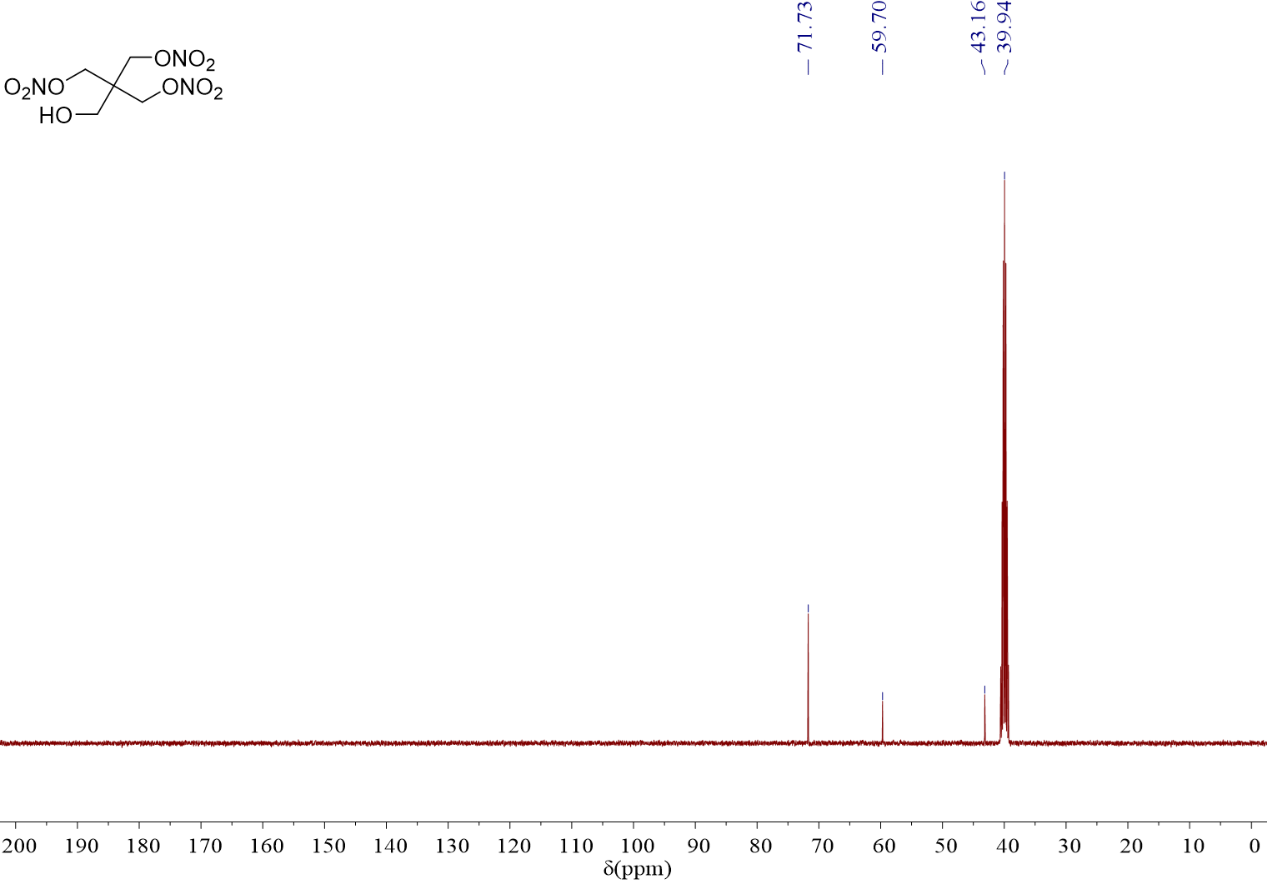
**

**Fig. S2.** ^13^C NMR of 3NO_3_-OH in DMSO-d6.


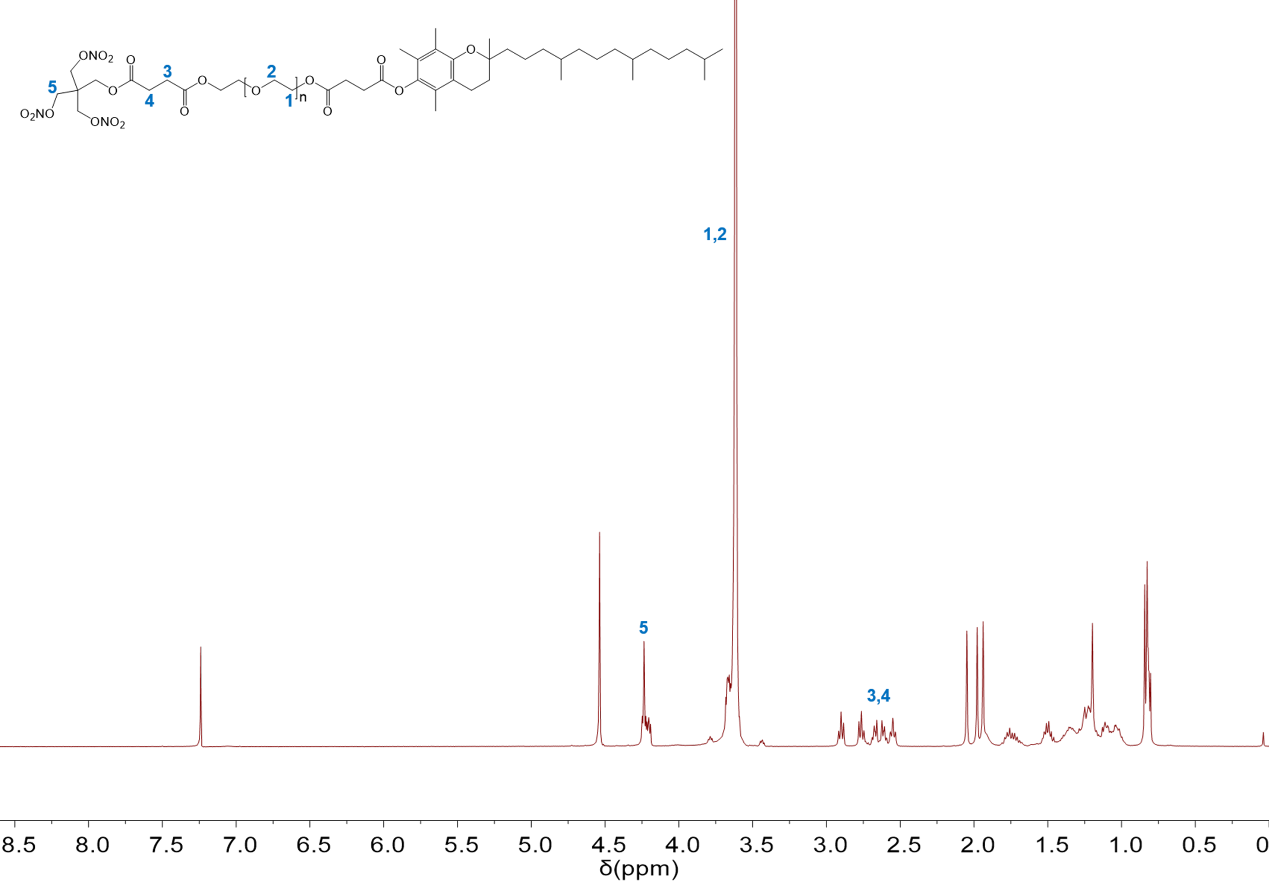


**Fig. S3.** ^1^H NMR of TPGS-3NO_3_ in chloroform-d. Characteristic peaks as marked in the graphs.


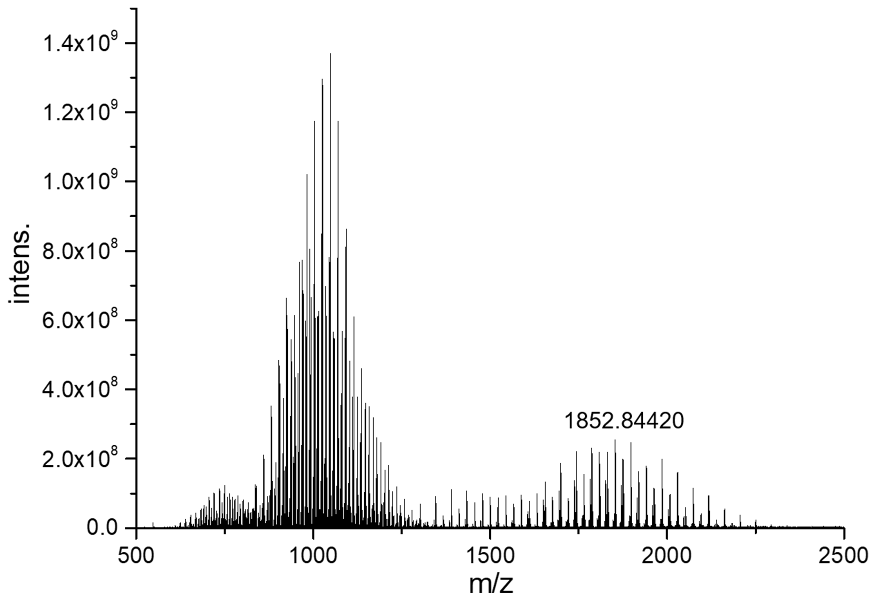


**Fig. S4.** HRMS of TPGS-3NO_3_.


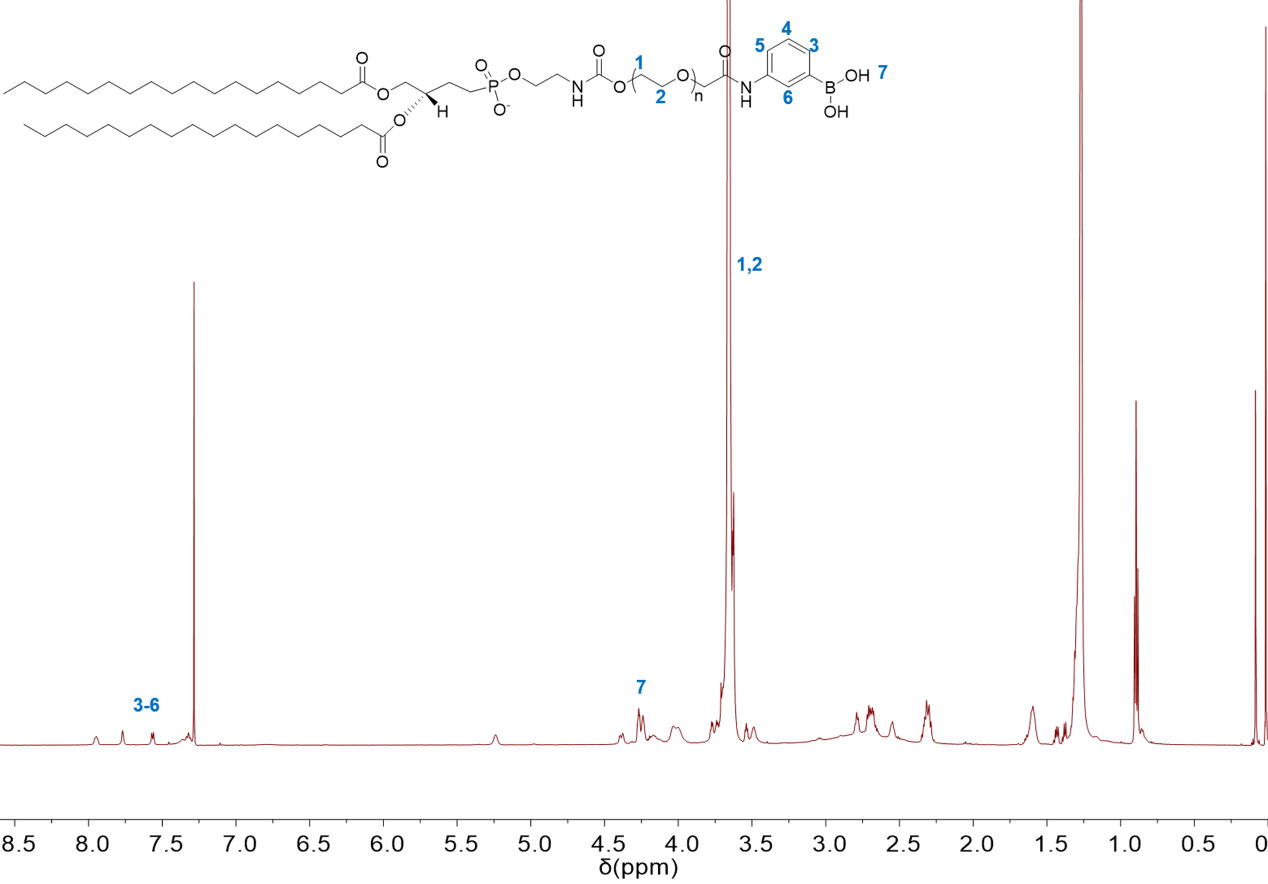


**Fig. S5.** ^1^H NMR of PBA-PEG2000-DSPE in chloroform-d. Characteristic peaks as marked in the graphs.


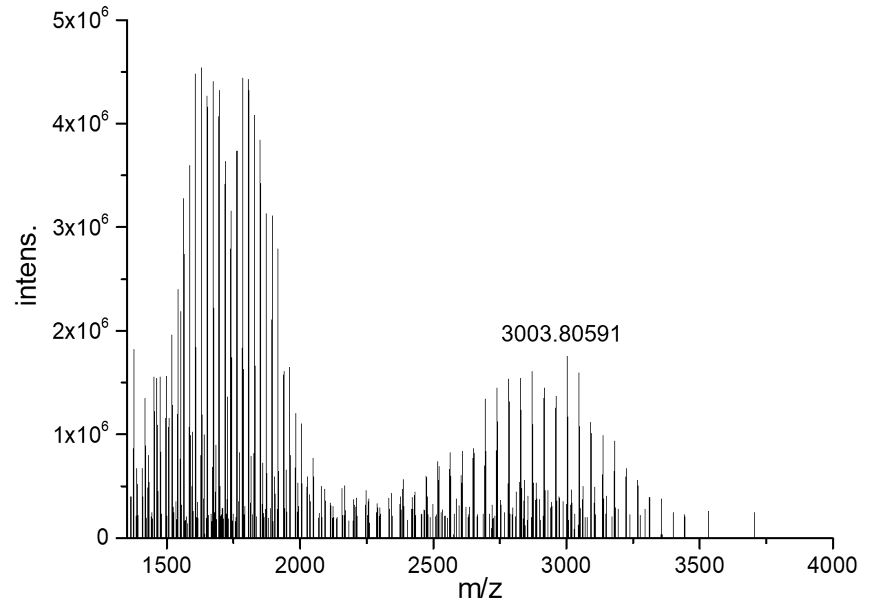


**Fig. S6.** HRMS of PBA-PEG2000-DSPE.


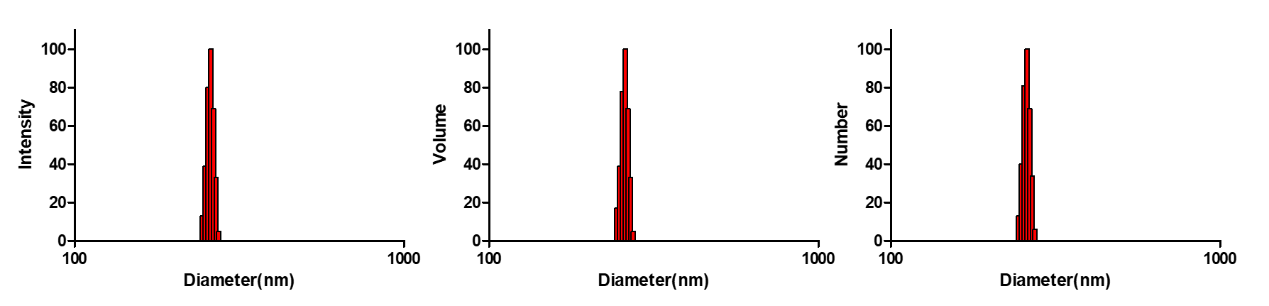


**Fig. S7.** Dynamic light scattering (DLS) size measurement of CTP/CDDP.


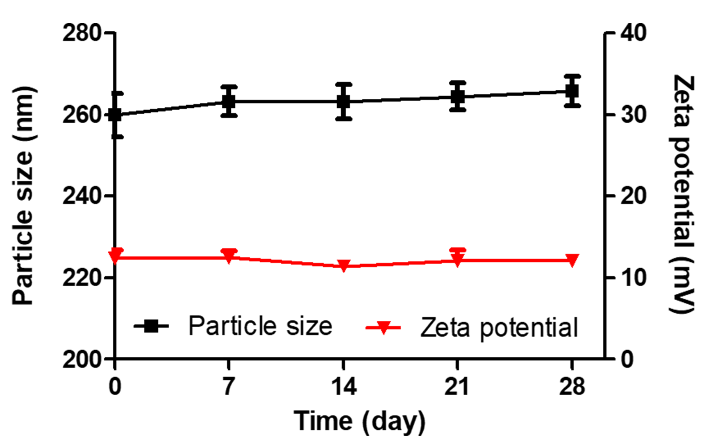


**Fig. S8.** Stability of CTP/CDDP at 4 ℃ for 4 weeks as measured by the particle size and zeta potential during storage. Data are expressed as mean ± SD (n = 3).


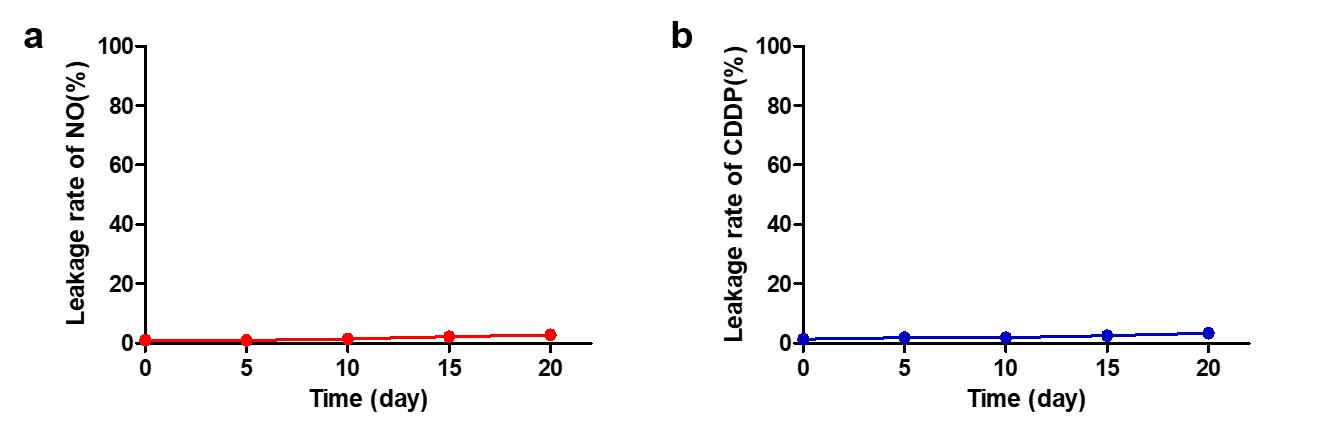


**Fig. S9.** Leakage rate of **(a)** NO and **(b)** CDDP from CTP/CDDP during storage at 4 ℃. Data are expressed as mean ± SD (n = 3).


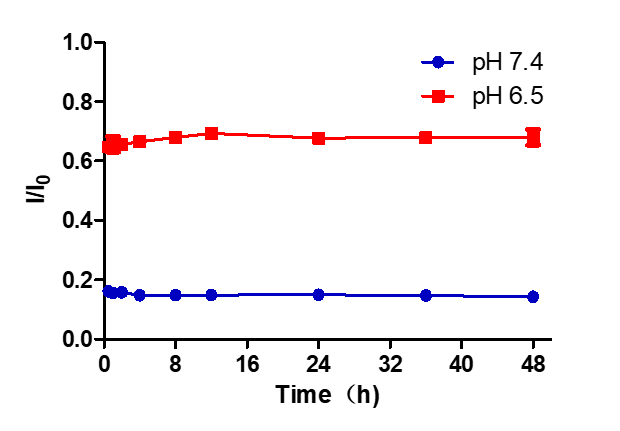


**Fig. S10.** The combination stability assay of PBA-COS conjugate at different time points.


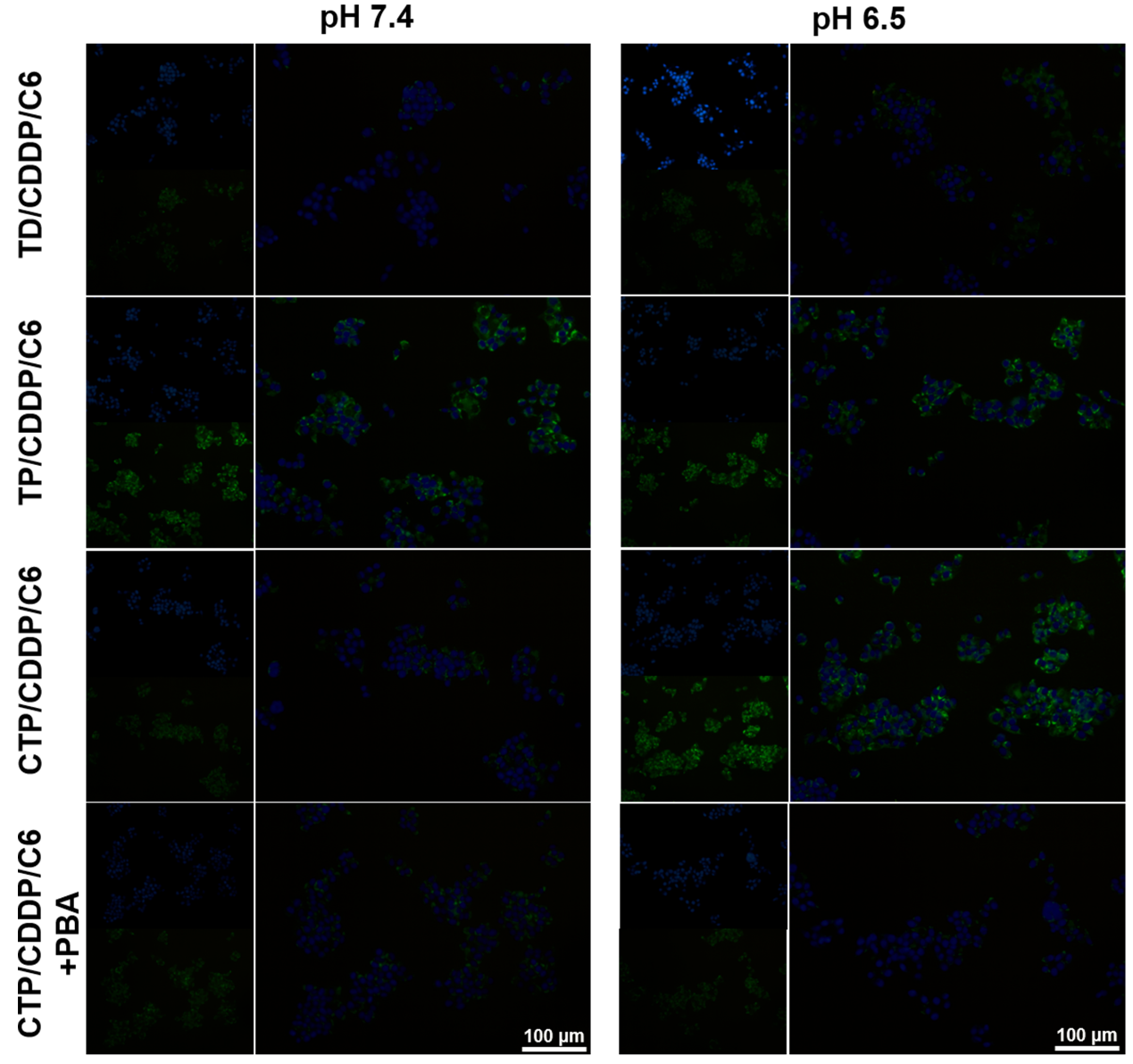


**Fig. S11.** Cellular uptake of MCF-7 cells after incubation with different drug formulations at pH 6.5 or 7.4 for 3 h.


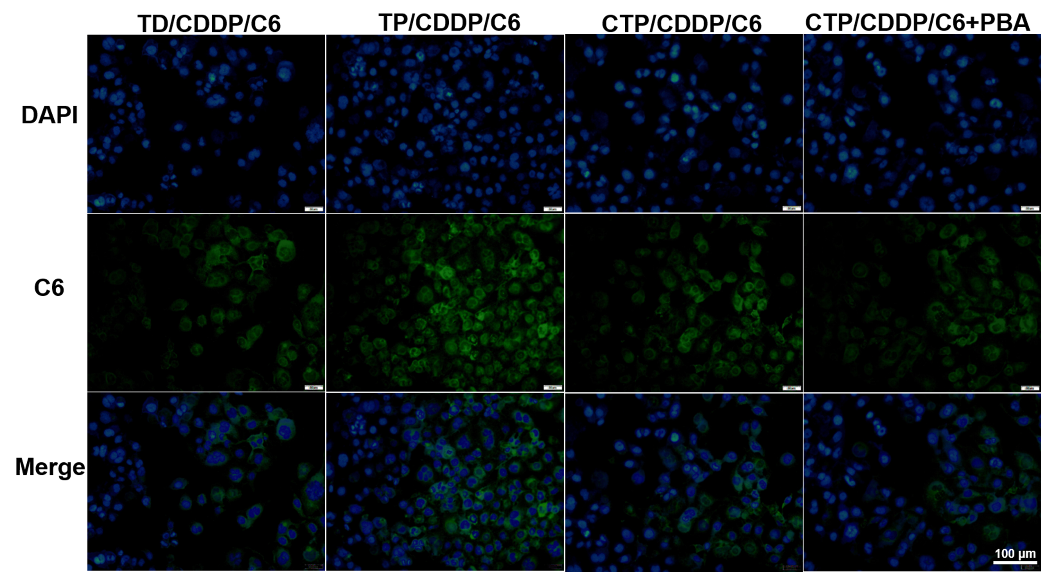


**Fig. S12.** Cellular uptake of L02 cells after incubation with different drug formulations at pH 7.4 for 3 h.


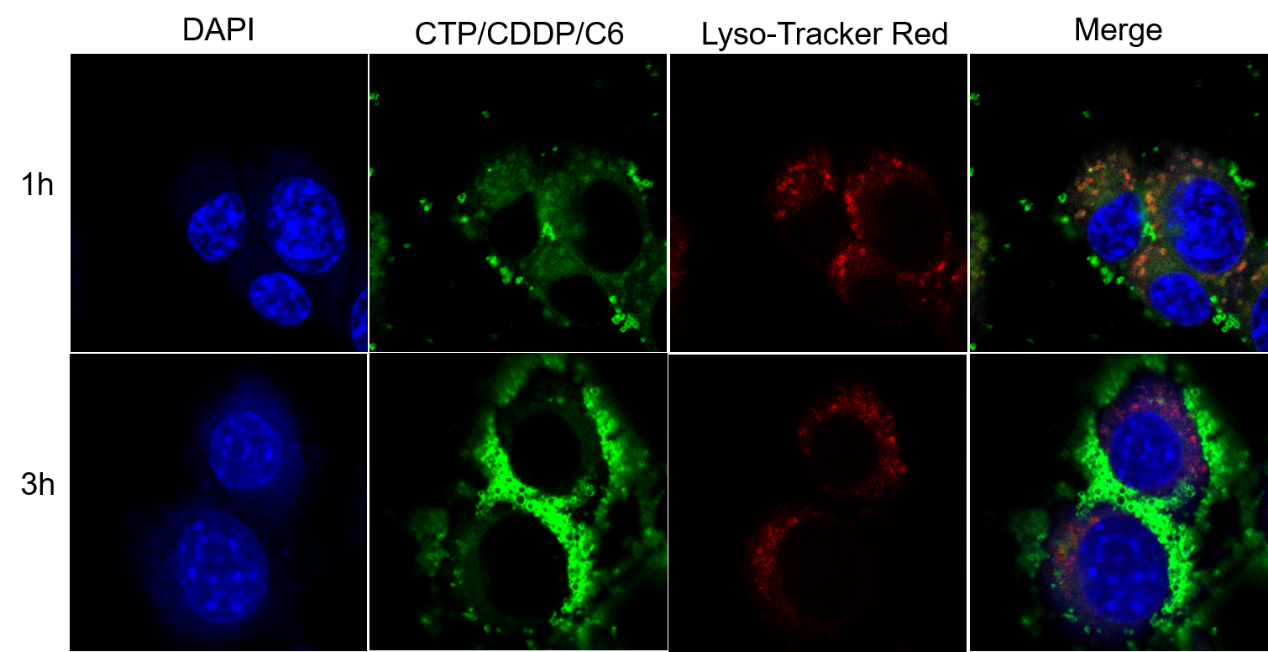


**Fig. S13.** CLSM images of 4T1 cells after incubation with CTP/CDDP/C6 for 1 h or 3 h.


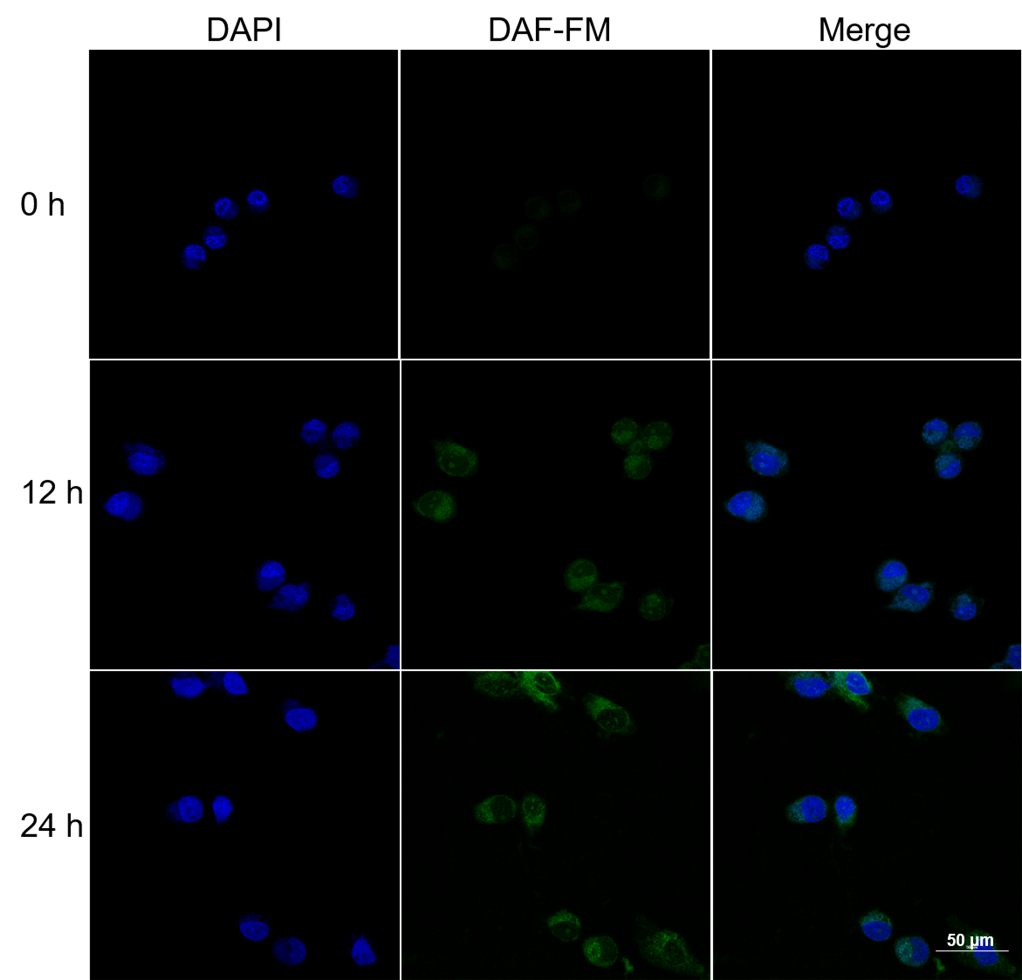


**Fig. S14.** Intracellular NO level in 4T1 cells after administration of CTP/CDDP for 0, 12 and 24 h.


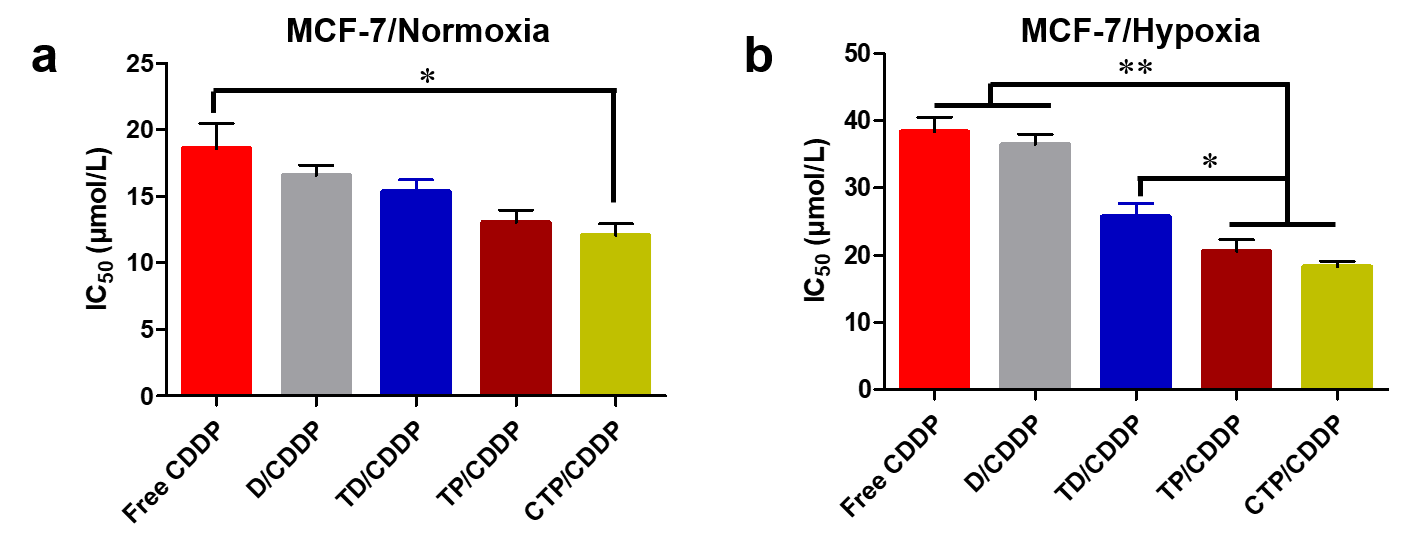


**Fig. S15.** *In vitro* anti-tumor effects of different drug formulations. IC_50_ was calculated to evaluate the cytotoxicity of various drug formulations after incubation with MCF-7 cells for 24 h under **(a)** normoxia and **(b)** hypoxia. Data are expressed as mean ± SD (n = 3), *p < 0.05, **p < 0.01.


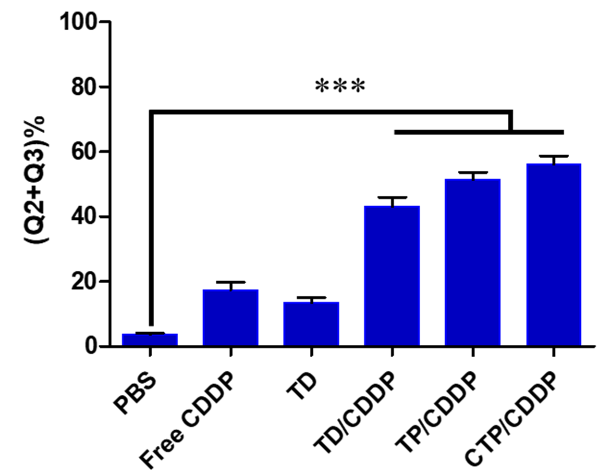


**Fig. S16.** The quantification of cell apoptosis. Data are expressed as mean ± SD (n = 3), ***p < 0.001.


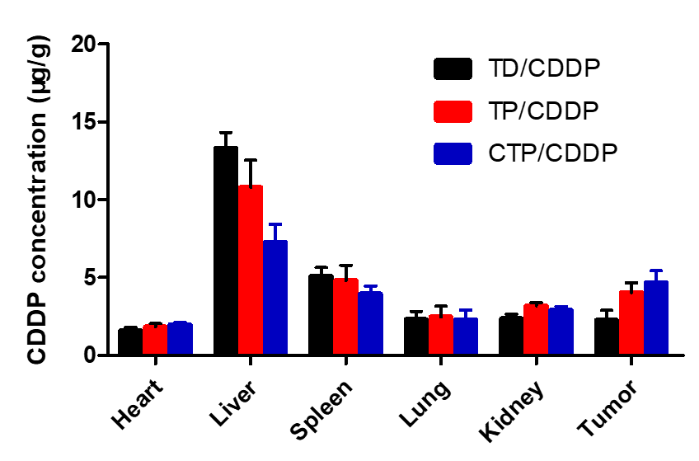


**Fig. S17.** CDDP biodistribution in tissues of 4T1 xenograft tumor-bearing mice at 12 h after intravenous injection. Data are expressed as mean ± SD (n = 5).


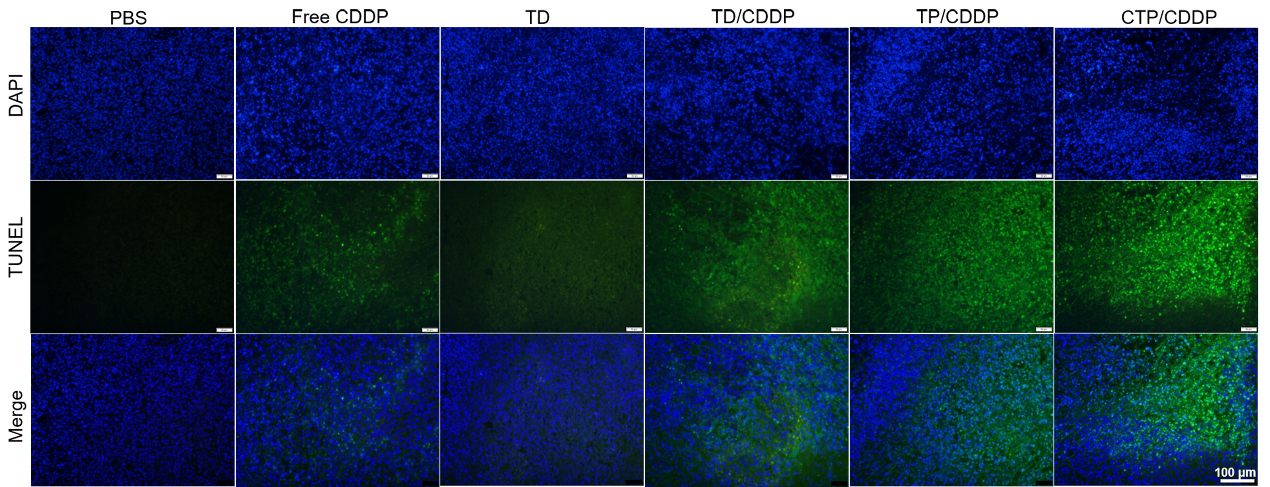


**Fig. S18.** *In vivo* anti-tumor efficiency of different drug formulations in mice bearing 4T1 xenograft tumors. Tumor apoptosis was determined by TUNEL assay.


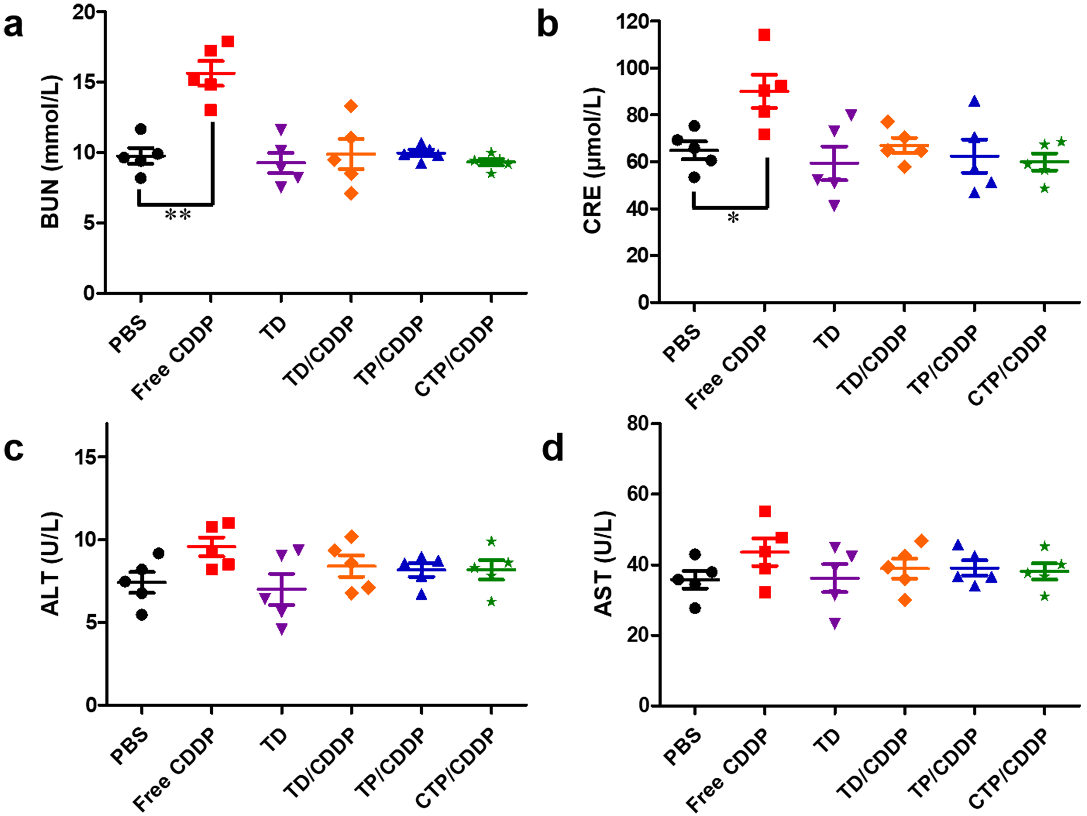


**Fig. S19.** Values of biochemical indexes of liver and kidney containing **(a)** BUN, **(b)** CRE, **(c)** ALT and **(d)** AST. Data are expressed as mean ± SD (n = 5), *p < 0.05, **p < 0.01.


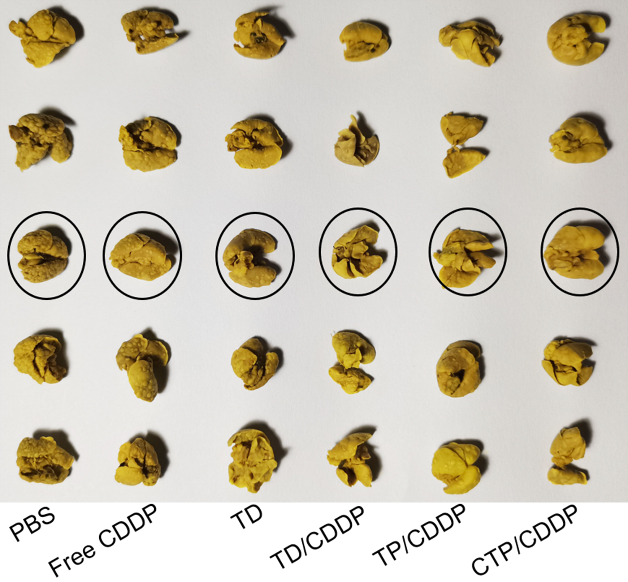


**Fig. S20.** *In* *vivo* anti-metastasis effect. Image of excised lung tissues at the end of the experiment (day 21 after tumor inoculation). Data are expressed as mean ± SD (n = 5).
